# Spreading depolarization triggers pro- and anti-inflammatory signalling: a potential link to headache

**DOI:** 10.1093/brain/awaf015

**Published:** 2025-01-17

**Authors:** Zeynep Kaya, Nevin Belder, Melike Sever-Bahcekapili, Şefik Evren Erdener, Buket Dönmez-Demir, Canan Bağcı, Merve Nur Köroğlu, Kaya Bilguvar, Turgay Dalkara

**Affiliations:** Institute of Neurological Sciences and Psychiatry, Hacettepe University, 06230 Ankara, Turkey; Institute of Neurological Sciences and Psychiatry, Hacettepe University, 06230 Ankara, Turkey; Institute of Neurological Sciences and Psychiatry, Hacettepe University, 06230 Ankara, Turkey; Institute of Neurological Sciences and Psychiatry, Hacettepe University, 06230 Ankara, Turkey; Institute of Neurological Sciences and Psychiatry, Hacettepe University, 06230 Ankara, Turkey; Institute of Neurological Sciences and Psychiatry, Hacettepe University, 06230 Ankara, Turkey; Department of Biostatistics and Bioinformatics, Institute of Health Sciences, Acıbadem Mehmet Ali Aydınlar University, 34684 Istanbul, Turkey; Departments of Neurosurgery and Genetics, Yale Program on Neurogenetics, Yale Center for Genome Analysis, Yale School of Medicine, New Haven, CT 06516, USA; Department of Medical Genetics, School of Medicine, Department of Translational Medicine, Genome Studies and Biostatistics and Bioinformatics, Institute of Health Sciences, Acurare, Acibadem Mehmet Ali Aydınlar University, 34684 Istanbul, Turkey; Institute of Neurological Sciences and Psychiatry, Hacettepe University, 06230 Ankara, Turkey; Departments of Neuroscience and Molecular Biology and Genetics, Bilkent University, 06800 Ankara, Turkey

**Keywords:** spreading depolarization, neuroinflammation, inflammation resolution, migraine, nuclear factor kappa B, fluorescence resonance energy transfer

## Abstract

Cortical spreading depolarization (CSD), the neurophysiological event believed to underlie aura, might trigger migraine headaches through inflammatory signalling that originates in neurons and spreads to the meninges via astrocytes. Increasing evidence from studies on rodents and migraine patients supports this hypothesis. The transition from pro-inflammatory to anti-inflammatory mechanisms is crucial for resolving inflammation. However, the resolution of inflammation in the context of CSD and migraine headaches remains poorly understood. This study aims to elucidate the progression of post-CSD inflammatory signalling and its resolution in neurons, astrocytes and microglia in mouse brains.

CSD was triggered optogenetically or by pinprick. High mobility group box 1 release, caspase-1 activation and cell-specific activation of nuclear factor kappa B (NF-κB) pairs, along with ensuing transcriptomic changes, were evaluated using immunofluorescence, western blotting, co-immunoprecipitation, fluorescence resonance energy transfer analysis and cell-specific transcriptomics.

Our findings indicate that after the initial burst, high mobility group box 1 release from neurons ceased, and caspase-1 activation, which peaked 1 h post-CSD, diminished within 3–5 h. This suggests that pro-inflammatory stimuli driving inflammatory signalling decreased within hours after CSD. Pro-inflammatory NF-κB p65:p50 pairs, along with anti-inflammatory cRel:p65 pairs, were detected in astrocyte nuclei shortly after CSD. However, 24 h post-CSD, the former had disappeared whereas the latter persisted, indicating a shift from pro- to anti-inflammatory activity in astrocytes. Pathway analysis of cell-specific transcriptomic data confirmed NF-κB-related pro-inflammatory transcription in astrocytes 1 h post-CSD, whereas no such activity was observed in neurons. Detailed transcriptomic analysis with Bayesian cell proportion reconstruction revealed that microglia exhibited transcriptional changes trending towards an anti-inflammatory profile, along with upregulation of several chemokines and cytokines (e.g. tumour necrosis factor). This suggests that microglia might play a role in supporting the inflammatory responses in astrocytes through the release of these mediators. The upregulation of genes involved in chemotaxis (e.g. *Ccl3*) and spine pruning (e.g. *C1q*) in microglia implies that microglia might contribute to synaptic repair, while inflammatory signalling in astrocytes could potentially modulate meningeal nociceptor activity through an extensive astrocyte endfeet syncytium abutting subarachnoid and perivascular spaces, although direct evidence remains incomplete. This nuanced understanding of the inflammatory response in CNS cell types highlights the intricate cellular interactions and responses to CSD.

Following a single CSD, distinct transcriptomic responses occur in neurons, astrocytes and microglia, driving inflammatory and anti-inflammatory responses, potentially contributing to headache initiation and resolution.

## Teaser

Cortical spreading depolarization (CSD)—an electrophysiological correlate of migraine aura—is thought to trigger headache through inflammatory signals in the brain and meninges. Using an intact mouse brain model, Kaya *et al.* explore the mechanisms that may suppress the neuroinflammatory response initiated by CSD.

## Introduction

Cortical spreading depression (CSD) is the neurophysiological correlate of migraine aura.^1^ The hypothesis proposing that ‘CSD can trigger migraine headaches through parenchymal inflammatory signalling, subsequently leading to sterile meningeal inflammation’ is gaining mounting experimental and clinical validation.^2-17^ Studies have revealed that the opening of pannexin-1 (Panx-1) channels in stressed neurons by CSD, the formation of the inflammasome complex, activation of caspase-1 and the subsequent release of interleukin-1 beta (IL-1β) and high mobility group box 1 (HMGB1) initiate pro-inflammatory nuclear factor kappa B (NF-κB) activation in astrocytes.^13,17-19^ Thus, it is likely that the pro-inflammatory transcription in astrocytes can play a part in CSD-induced headache.^5,14,18,20-25^ This hypothesis is supported by findings indicating that inhibition of NF-κB suppresses CSD-induced middle meningeal artery dilatation, a surrogate marker of trigeminovascular activation.^18^ Additionally, disrupting astrocyte function with gliatoxins reduces CSD-evoked dural nociceptor sensitization, as demonstrated electrophysiologically.^14^ This perspective is reinforced further by numerous studies reporting that astrocytes release pro-inflammatory agents, including ATP, prostanoids and cytokines/chemokines (reviewed by Verkhratsky and Nedergaard^26^ and by Carneiro-Nascimento and Levy^27^). A recent study showed the presence of inflammatory mediators with proteomic analysis of post-CSD CSF.^28^ Corroborating these hypotheses with experimental data on rodents, recent PET studies conducted on patients suffering from migraine with aura, following the injection of [^11^C]PBR28 (a molecule taken up by glial cells during inflammation), revealed tracer uptake in both parenchymal and meningeal regions.^8,11^ Intriguingly, tracer uptake was registered simultaneously in the affected occipital (aura) cortex and the overlying dura in some patients. This finding lends support to the concept that CSD-induced parenchymal inflammatory signalling can propagate to the meninges, triggering meningeal inflammation and, consequently, headache.^11^

The primary purpose of inflammation is to maintain tissue homeostasis.^29^ To achieve this goal, the inflammatory process must be regulated by various mechanisms to prevent undesired tissue damage, a complex process known as the resolution of inflammation.^29,30^ In this process, which has been characterized for several inflammatory disorders, inflammatory cells infiltrating the tissue, if any, are eliminated with the help of apoptosis, or the resident cells such as microglia return to their inactive forms and contribute to the resolution process by releasing anti-inflammatory mediators.^29,30^ Generally, the release of the pro-inflammatory molecules (e.g. IL-1β) is replaced with anti-inflammatory ones (e.g. interleukin-10). The resolution process typically follows inflammation, although it may sometimes commence simultaneously with inflammation. In instances where inflammation does not resolve completely, chronic inflammatory disorders can emerge.^29-31^ It is probable that anti-nociceptive neural networks within pain pathways and the attenuation of sensitization mechanisms at nociceptive synapses play a role in terminating headaches and determining their variable duration. However, the resolution of the inflammatory response might also contribute to this phase by halting the driving force behind nociceptive activity.^32^ Although the mechanisms suppressing inflammation during the resolution of pathological inflammation in the brain are generally understood, there is no specific study addressing migraine headaches or CSD in this context.^33-37^

The NF-κB transcription factor family operates through the assembly of five distinct subunits (p65, p50, cRel, RelB and p52) in pairs.^38^ Depending on the specific subunit pairs, NF-κB either promotes the expression of pro-inflammatory molecules (e.g. p65:p50) or anti-inflammatory ones (e.g. cRel:p50).^35-37,39-41^ Our laboratory has previously demonstrated that the pro-inflammatory subunit p65 plays a crucial role in the inflammatory response initiated by CSD.^13,18,19^ We hypothesize that following CSD, the active subunit pairs might undergo a temporal shift, transitioning from inflammation-initiating pairs (such as p65:p50) to resolving pairs (e.g. cRel:p50). This potential alteration could contribute to the attenuation of neuroinflammatory signalling in the parenchyma. Moreover, the binding of inhibitor kappa B (IκB) to active pairs within the nucleus could represent an additional effective mechanism for resolving inflammation.^38^ Additionally, the release of HMGB1, which activates the NF-κB system, might not be long lasting. Likewise, over time, inflammasome/caspase-1 activity in neurons might diminish, resulting in reduced release of pro-inflammatory molecules, such as IL-1β. This reduction could contribute to resolution of inflammation by decreasing the stimulation of the pro-inflammatory NF-κB pathway in astrocytes. In this study, we explore these potential mechanisms that might suppress the neuroinflammatory response initiated by CSD in the intact mouse brain. We aim to identify proteins and transcripts involved in these processes.

## Material and methods

### Animals and induction of CSD

Animal housing, care, and application of experimental procedures were all performed per institutional regulations as approved by the Hacettepe University Animal Experiments Local Ethics Committee (approval numbers: 2017/10-5 and 2018/30-1). Adult male Swiss mice (*n* = 66) and adult female Thy1-ChR2-YFP mice (*n* = 12), which express the light-activated ion channel channelrhodopsin-2 fused to yellow fluorescent protein under the control of the mouse thymus cell antigen 1 (*Thy1*) promoter^42^ (stock #007612, Jackson Laboratories), were used. All results, except for the transcriptomic analyses, were obtained from Swiss mouse brains after CSDs induced by pinprick under anaesthesia. To avoid the well-known effects of anaesthesia and surgical manipulations on gene transcription, optogenetic stimulation of the cortex in unanaesthetized, freely moving Thy1-ChR2-YFP mice was used for the transcriptomic study. For the details of protocol, please refer to Supplementary material. We used Swiss male mice to maintain consistency with the previously published data.^18,19^ However, we chose to use female Thy1-ChR2-YFP mice because migraine is more common in women, and transcriptomic changes can be influenced significantly by sex, unlike post-translational mechanisms, such as HMGB1 release, caspase activation and NF-κB translocation, where the impact of sex is likely to be much more modest.

### Immunofluorescent labelling

The sections were immunostained with antibodies against HMGB1, Iba1, activated caspase-1, NeuN, p65, cRel, p50 and IκB-α, followed by labelling with appropriate secondary antibodies. Double immunolabellings (p65–p50, p65–cRel, p50–cRel, p65/cRel/IκB–NeuN and p65/cRel/p50–Iba1) were carried out with incubation of primary antibodies either simultaneously or consecutively. All sections were examined under a laser scanning confocal microscope (SP8, Leica GmbH) with appropriate filter sets. For statistical analyses based on cell counting, multiple brain sections were examined consistently from each animal, minimizing technical variability. For the details of protocols, please refer to Supplementary material and Supplementary Table 1.

### Fluorescence resonance energy transfer

A three-dimensional spectral unmixing approach was used to evaluate fluorescence resonance energy transfer (FRET) efficiency between donor and acceptor fluorophores of secondary antibodies used for immunolabelling of NF-κB subunits. Donor-only stained and acceptor-only stained brain sections were used for the acquisition of reference spectra. All samples were imaged in the lambda-scan mode of Leica LasX software, using 488 and 552 nm lasers separately, with 5-nm-wide windows of 3 nm steps within the range of 495–695 nm, excluding the band that corresponds to the excitation laser. The RT 15/85 beam splitter was used to avoid spectral losses filtered out by dichroic mirrors. Fluorescence emission was collected with the hybrid detector with more stable efficiency across the emission bandwidths. Regions of interest measuring 100 μm × 100 μm were scanned with 1024 × 1024 pixels, using a ×63 oil-immersion objective (NA: 1.3). Eight-bit lambda stacks were acquired from donor-only reference sections, acceptor-only reference sections and donor–acceptor double-stained sections. The data were processed with the FRETTY plugin of FIJI.^43,44^ In short, the emission spectrums of the double-stained sample at two different excitation wavelengths were compared with the spectra from donor-only or acceptor-only stained samples, accounting for the excitation of the acceptor fluorophore directly with the donor excitation wavelength. In plugin parameters, quantum yields were entered as 0.92 and 0.69 for Alexa488 and Alexa568 fluorophores, respectively. The pixel threshold was selected as 50 for the generation of FRET-efficiency (E-value) maps. The final presented images were Gaussian filtered (σ =1 pixel) for removal of noise and better clarity.

### Nuclear protein extraction

For the extraction of nuclear and cytoplasmic proteins, the instructions of the commercial kit manufacturer (NE-PER Nuclear and Cytoplasmic Extraction Kit, 78833, Thermo Fisher Scientific) were followed, with a few modifications. After cervical dislocation, the brain was quickly removed and divided into ipsilateral and contralateral cortices on ice. Phosphatase and protease inhibitor cocktail (1:100) was added to the first solution of the kit. Each hemicortex was dissected with a blade-type homogenizer in the first solution until no visible fragments remained. The following steps were performed as instructed by the manufacturer. The obtained samples were stored at −80°C until western blotting.

### Western blotting

Hemicortices were lysed in radioimmunoprecipitation assay buffer. The protein concentration of all lysates was determined by a bicinchoninic acid assay (23227, Thermo Fisher Scientific). Equal amounts of protein were loaded on gels and subsequently transferred to polyvinylidene difluoride membranes. For details of the protocol, please refer to Supplementary materials. To analyse the nuclear and cytoplasmic fractions of cRel, p65 and IκB, the integrated density values of the bands representing the cytoplasmic fraction were compared with β-tubulin as a loading control, and the values of the bands representing the nuclear fraction were compared with histone-3 (nucleus-specific structural protein). The nuclear/total protein ratio was calculated by comparing the amount of nuclear protein to the sum of nuclear and cytoplasmic proteins. For full-length blot images, please refer to Supplementary Figs 4-6.

### Tissue dissociation and isolation of neurons by magnetic-activated cell sorting

Given that physical stress, such as pushing cells through microchannels under pressure during cell dissociation, can induce inflammatory transcription,^45^ we opted for gentle cell separation techniques combined with Bayesian single-cell deconvolution analysis to minimize procedure-related cellular stress. The cortex and subcortical structures were separated on ice with a spatula under a microscope and tissue dissociation was conducted using the Adult Brain Dissociation Kit (130-107-677, Miltenyi Biotec) following the manufacturer’s instructions, with slight modifications. Mouse neurons were negatively selected by depleting non-neuronal cells using the Neuron Isolation Kit (130-115-390, Miltenyi Biotec) following the manufacturer’s guide (for a detailed illustration of the experimental steps, visit https://www.miltenyibiotec.com/UN-en/products/neuron-isolation-kit-mouse.html and https://www.miltenyibiotec.com/UN-en/resources/macs-handbook/macs-technologies/cell-separation/magnetic-cell-separation.html). For details of the protocols, please refer to Supplementary materials. This method achieves high neuronal purity (98.6%–99.9%) and >90% cell viability, with minimal contamination from non-neuronal cells, as confirmed by our RNA-sequencing data (Supplementary Table 2).

### RNA isolation, quantification, quality–integrity determination, sequencing and transcriptomic analysis

RNA was isolated with TRIzol (T9424, Sigma Aldrich) in accordance with the manufacturer’s protocol. Quantification and determination of the purity of the isolated RNAs were performed using a NanoDrop™ 1000 Spectrophotometer (Thermo Fisher Scientific). For details of the isolation protocol, please refer to Supplementary material.

The RNA samples extracted from four groups (neuronal and non-neuronal cells obtained from sham surgery and CSD groups) were sent to BGI Genomics Inc. (Wuhan, China) for RNA sequencing and analysis. Briefly, the quality and integrity of isolated RNAs were analysed using the Agilent 2100 Bioanalyzer. All samples except one (6.4) exhibited a RNA integrity number score >7, indicating sufficient quality for subsequent library construction and sequencing.^46,47^ We also observed a high Pearson correlation coefficient among three brain samples in each group, as illustrated in Supplementary Table 3. Additionally, all samples in our study had Q30 values (percentage of sequenced bases that have a predicted quality score of 30 or more) of >85%.^48^ RNA sequencing was carried out using the BGISEQ500 platform (BGI Genomics), and transcriptomic expression analysis was performed on 12 samples. The HISAT2 program (v.2.0.1) was used for alignment against the reference genome (Reference Genome Version: GCF_000001635.26_GRCm38.p6), and Bowtie2 software (v.2.2.5) was applied to align the clean reads to the reference genes. Differential expression analysis was conducted using the DESeq2 algorithm, applying criteria of log2(fold change) ≥ 0.58 and *P*-value < 0.05. The single-cell transcriptome reference matrix including read counts of both neuronal and non-neuronal cells from multiple cortical areas of mice samples was preprocessed based on cell types and subclass information obtained from the provided metadata. Read counts of CSD and sham groups were used for the deconvolution of cell type composition and the gene expression profile of each cell type using BayesPrism (v.2.0) with the preprocessed reference read counts. Subsequently, comprehensive analyses, including heatmap generation, Gene Ontology (GO; geneontology.org), Kyoto Encyclopedia of Genes and Genomes (KEGG; http://www.genome.jp/kegg/) and Gene Set Enrichment Analysis (GSEA), were performed. These analyses were carried out on the online bioinformatic platform, Dr. Tom (https://biosys.bgi.com/), provided by BGI, which integrates various published software for data mining. The fold-change values provided in Supplementary Table 2 represent neurons relative to non-neuronal cells; those in Supplementary Table 6 are astrocyte specific relative to microglia specific, and the values in Supplementary Tables 7 and 8 compare CSD with sham surgery.

### Statistical analysis

After determining whether the data were distributed normally or not, one-way ANOVA or the Kruskal–Wallis test was applied for comparison of more than two independent groups. *Post hoc* analysis was performed according to the homogeneity of data (Levene test) with Tukey’s test or the Tamhane test following ANOVA. Pairwise comparisons were made with the Mann–Whitney U-test after the Kruskal–Wallis test. Bonferroni correction was applied to determine the significance level during *post hoc* testing. ANOVA linear contrast test was used for trend analysis.

## Results

### Termination of caspase-1 activity

**Figure 1:**
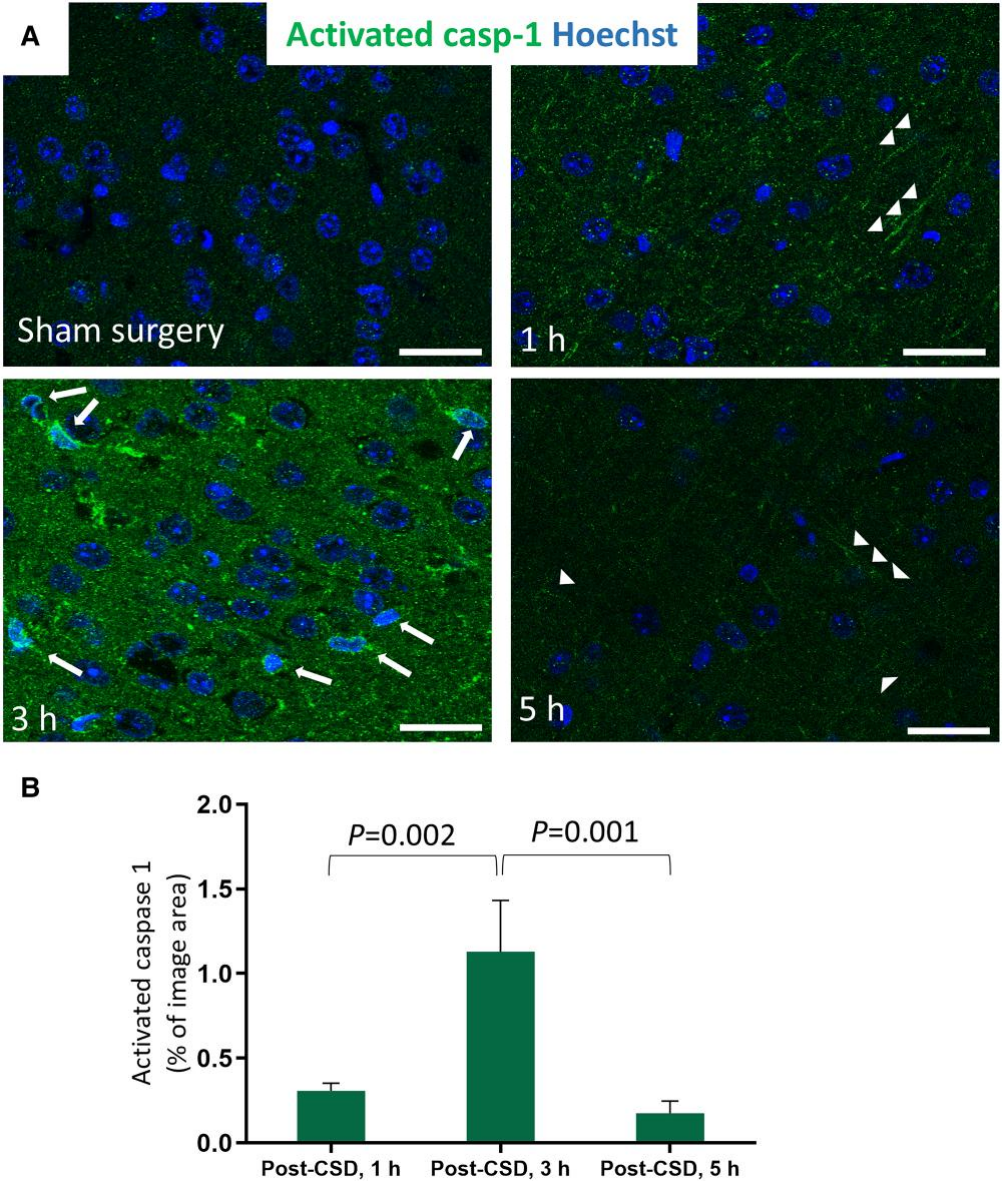
**Caspase-1 activity subsides within 5 h post-CSD.** (**A**) Different patterns and intensities of activated caspase-1 are observed in the ipsilateral cortex at 1, 3 and 5 h post-CSD and 3 h after sham surgery. Slight labelling in the dendrites (arrowheads) is visible 1 h post-CSD, becoming more pronounced in both the soma (arrows) and neuropil at 3 h. By 5 h post-CSD, activated caspase-1 is predominantly observed in the processes (arrowheads), albeit faintly. Caspase-1 activity is nearly absent following sham surgery. Scale bar: 25 μm. (**B**) The proportion of the activated caspase-1 pixels to the total area increases significantly at 3 h post-CSD (*P* = 0.001, Kruskal–Wallis test, *n* = 3 mice for each time point). Columns and error bars represent the mean and standard error of the mean; *P*-values above the bars correspond to two-group comparisons with Bonferroni correction. casp-1 = caspase-1; CSD = cortical spreading depolarization.

We previously showed the activation of caspase-1 in neurons shortly after CSD.^18^ Given that termination of the inflammasome, and consequently caspase-1 activity, is a crucial step in resolution of inflammation,^49,50^ we monitored caspase-1 activity post-CSD by detecting the active cleaved form of caspase-1 (p20) using immunofluorescent labelling and western blotting. Brain samples were collected at 1, 3 and 5 h post-CSD (*n* = 3 mice for each time point). At 1 h post-CSD, punctate caspase-1 p20 labelling was observed in the extensions and soma of neurons (Fig. 1A). By 3 h post-CSD, punctate labelling was largely replaced by diffuse and more intense labelling in both extensions and soma (Fig. 1). The area of activated caspase-1 pixels was significantly higher at 3 h post-CSD compared with 1 and 5 h (*P* = 0.001, Kruskal–Wallis test). This change might suggest that caspase-1 p20 monomers were dissociated from the inflammasome complex and dispersed into the cytoplasm, allowing increased antibody access. Conversely, at 5 h post-CSD, the caspase-1 p20 signal was barely detectable. Given that the most prominent labelling was observed 3 h post-CSD, control brains were examined 3 h after sham surgery. However, no similar labelling was detected in these control samples.

We also evaluated caspase-1 activation through western blotting, detecting both the non-cleaved inactive form (procaspase-1) and the active form (p20) of the enzyme in the cortex at 3 h post-sham surgery and 1, 3 and 5 h post-CSD (Supplementary Fig. 1A). Notably, the brains were removed without *in situ* fixation for western blotting, unlike immunofluorescent labelling, and caspase-1 is inevitably activated owing to brain anoxia/ischaemia during the brain extraction and lysate preparation.^51,52^ We observed that the active enzyme ratio in cortical lysates was 11% ± 3.0% at 1 h post-CSD, showing a decreasing trend over 5 h (*P* = 0.06; *n* = 3; Supplementary Fig. 1B). However, given that the p20 signal in western blotting is confounded by the tissue extraction process and diluted by caspase-1 from non-neuronal cells, the immunofluorescent labelling of *in situ* fixed brains appears to be the most accurate method for assessing caspase-1 activity over time. This method suggests the termination of caspase-1 activity within 3–5 h post-CSD.

### Termination of HMGB1 release

**Figure 2:**
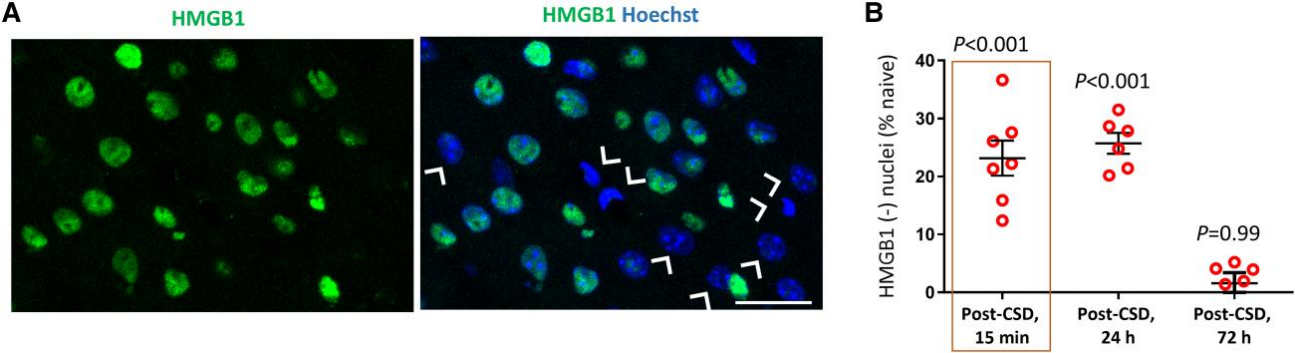
**HMGB1 replacement 72 h post-CSD.** (**A**) The representative immunofluorescent images illustrate the nuclei (identified by Hoechst labelling) releasing their HMGB1 content (arrowheads). These images are maximum projections of confocal microscopy *z*-stacks. Scale bar: 10 μm. **(B**) The ratio of HMGB1-immunonegative nuclei (expressed as a percentage of naïve Hoechst-positive nuclei) exhibits no significant increase from 15 min to 24 h post-CSD (*P* < 0.001 for both time points, compared with naïve using the Mann–Whitney U-test). However, at 72 h, the ratio of HMGB1-immunonegative nuclei is comparable to naïve brains (*P* = 0.99, compared with naïve using the Mann–Whitney U-test). This suggests that HMGB1 is rapidly released after CSD and subsequently replaced within 24–72 h (*n* = 3 mice for each time point). Lines and error bars represent the mean and standard error of the mean. The data from 15 min post-CSD are boxed to denote their previous use by Kaya *et al.*^19^ under the terms of the Creative Commons Attribution License (https://creativecommons.org/licenses/by/4.0/). CSD = cortical spreading depolarization; HMGB1 = high mobility group box 1.

In addition to IL-1β generated by caspase-1 activity, HMGB1 is another pro-inflammatory mediator released from neurons after CSD.^2,4,13,17-19^ Consequently, we investigated the temporal pattern of HMGB1 release post-CSD. Despite the expression of HMGB1 in the nuclei of all brain cells, commercial antibodies sometimes fail to label HMGB1 in certain glia.^53^ For this, in naïve animals, an average of 83% ± 1.8% of cells were labelled for HMGB1 in the somatosensory cortex, which was considered the baseline value to assess CSD-induced changes (*n* = 3 mice). Given consistent evidence that HMGB1 release occurs exclusively from neurons during CSD,^2,13,17-19^ we assessed the ratio of HMGB1-negative nuclei relative to all cells marked with the nuclear marker Hoechst in the naïve brain, as well as at 15 minutes, 24 hours, and 72 hours post-CSD (Fig. 2). The proportion of HMGB1(+) nuclei decreased from 83% to 63% within 15 min post-CSD, corresponding to a 23% reduction relative to the baseline (Supplementary Table 4). Given that this value did not increase further at later time points, we concluded that HMGB1 release was limited to the period of intense depolarization during CSD. In line with a study demonstrating the slow turnover of HMGB1 protein and its partial return 24 h post-CSD,^2^ our results indicate complete replacement of nuclear HMGB1 within 72 h.

### Differential activation of NF-κB subunits in neuronal and non-neuronal cells

**Figure 3:**
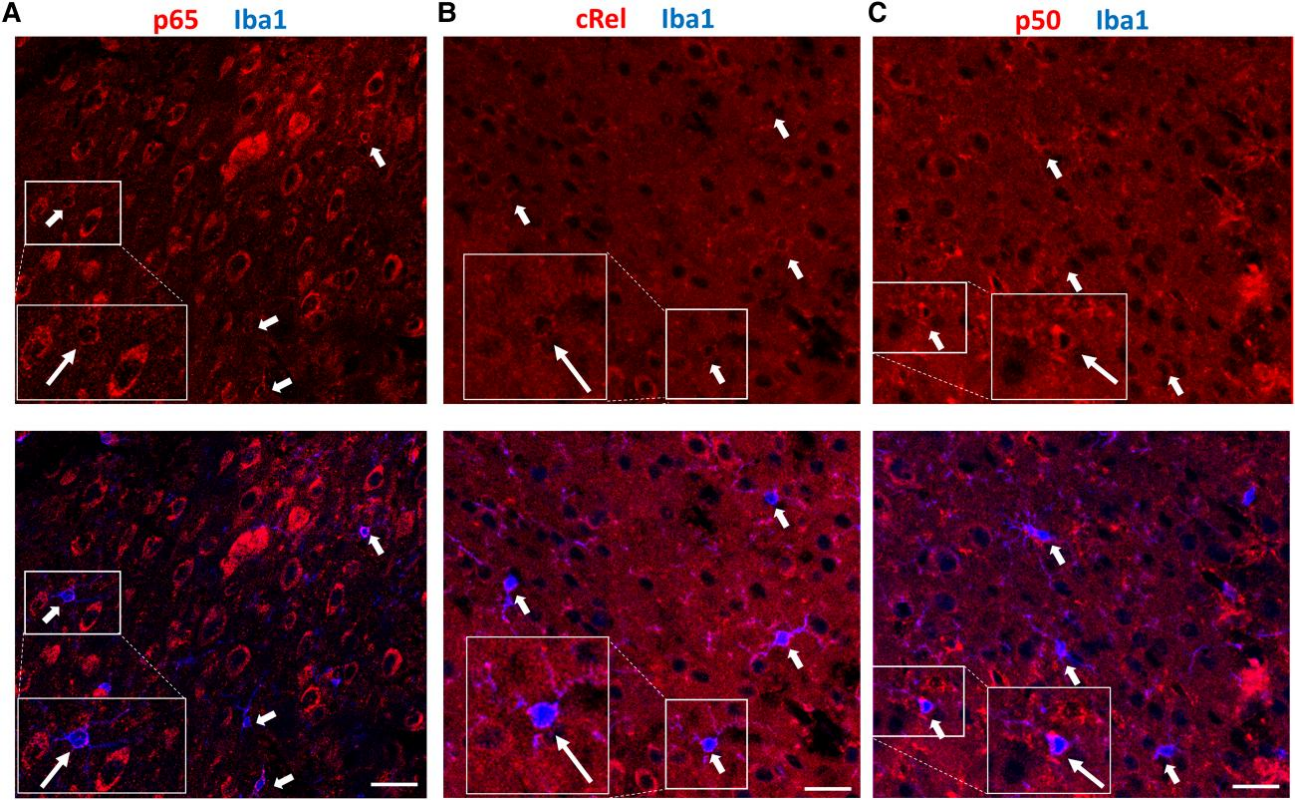
**NF-κB is not active in microglia 24 h post-CSD.** Double immunofluorescent labelling of NF-κB subunits p65 (**A**), cRel (**B**) and p50 (**C**) together with the microglia marker Iba-1 show the absence of NF-κB activation in microglia 24h post-CSD. Microglia (blue in *bottom row*), identified by thin processes and small, round cell bodies indicative of a resting state morphologically, exhibit no NF-κB subunits in their nuclei. Arrows highlight microglia, and magnified insets depict microglia with only cytoplasmic (inactive) NF-κB subunits in red (*n* = 3 mice). Scale bar: 25 μm. CSD = cortical spreading depolarization; NF-κB = nuclear factor kappa B.

**Figure 4:**
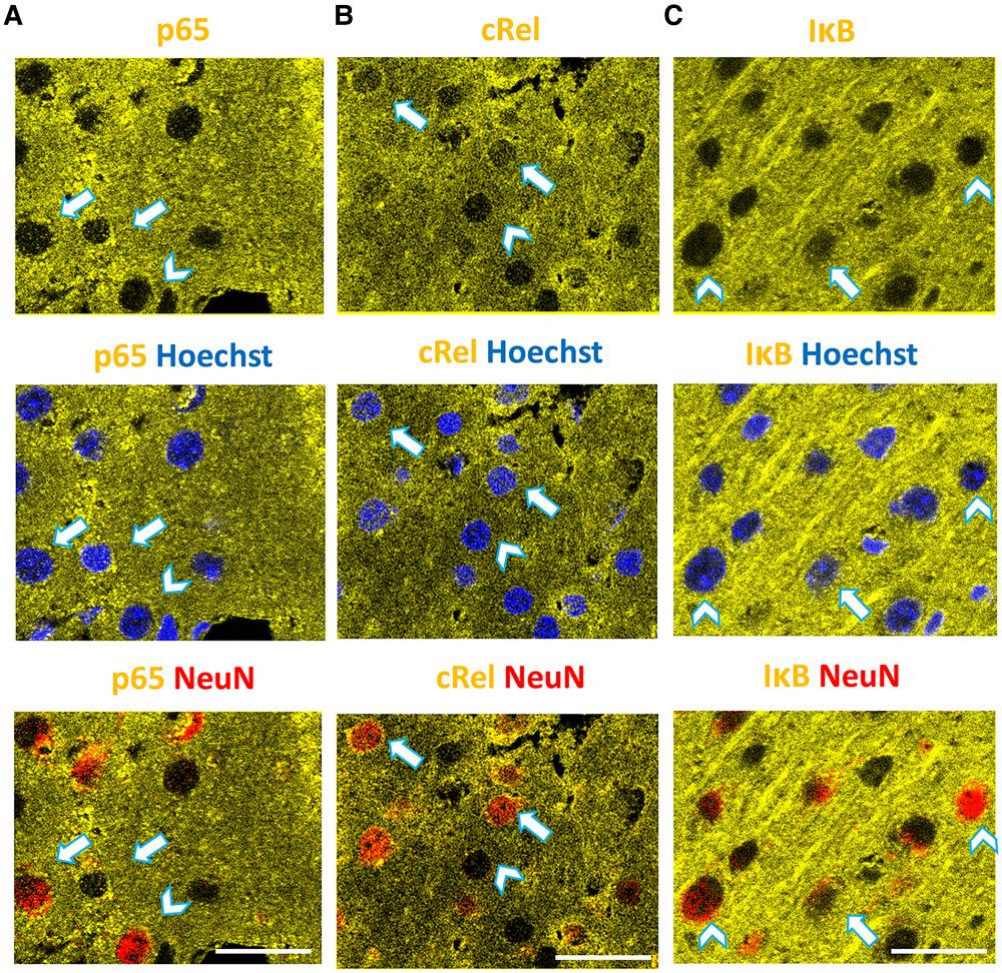
**Translocation of NF-κB subunits and IκB to the nucleus in neurons and non-neuronal cells.** p65 and cRel, pro- and anti-inflammatory subunits of NF-κB, along with their inhibitor IκB, undergo translocation to the nucleus in a cell type-dependent manner. In representative images from the ipsilateral cortex, the presence of cytoplasmic (arrowheads) and nuclear (arrows) p65 (**A**), cRel (**B**) or IκB (**C**) immunoreactivity (yellow) is observed in NeuN^+^ neurons (red) and non-neuronal cells. The *top row* depicts the distribution of immunoreactivity in nuclear, perinuclear and dendritic compartments. The *middle row* displays Hoechst-labelled nuclei (blue), and the *bottom row* specifically highlights neuronal nuclei (red). Images of p65 and cRel were captured 5 h post-CSD, as both are active in non-neuronal cells and neurons. In contrast, IκB images were captured 15 min post-CSD, as nuclear IκB is not observed in neuronal nuclei at 5 h (see Fig. 5). For quantitative insights into nuclear translocation before and after CSD, refer to the detailed data presented in Fig. 5. Scale bar: 10 μm. CSD = cortical spreading depolarization; IκB = inhibitory kappa B-α; NF-κB = nuclear factor kappa B.

**Figure 5:**
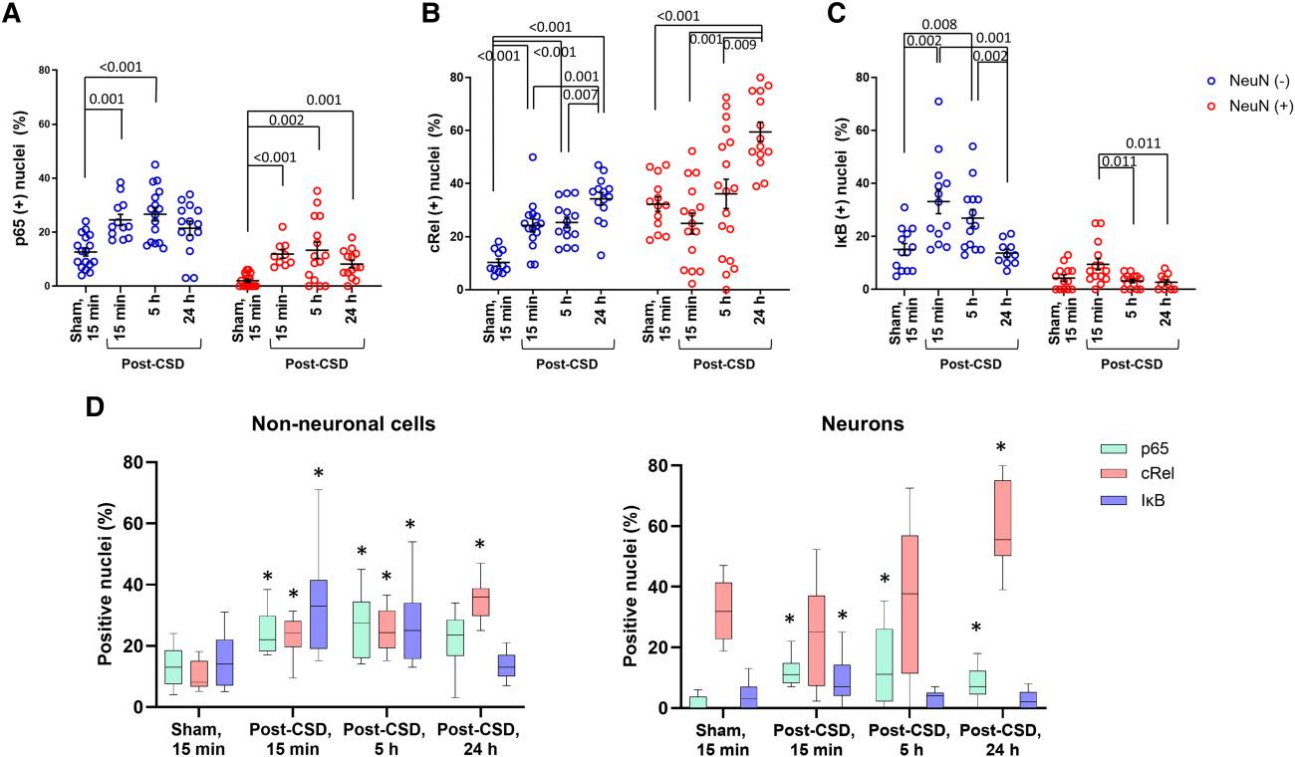
**Activation dynamics of NF-κB subunits and IκB in neurons and non-neuronal cells post-CSD.** Individual plots (**A**–**C**) and summary plots (**D**) illustrate the time- and cell type-dependent activation patterns of NF-κB subunits p65 and cRel, representing pro- and anti-inflammatory responses, and their inhibitor IκB. The analyses cover time points 15 min after sham surgery and 15 min, 5 and 24 h post-CSD. (**A**) In comparison to sham surgery, p65 was rapidly activated after CSD in NeuN(−) and to a lesser extent in NeuN(+) cells, decreasing after 24 h (Kruskal–Wallis test for NeuN(+) cells, *P* < 0.001; one-way ANOVA for NeuN(−) cells, *P* < 0.001). (**B**) In non-neuronal cells, cRel was immediately translocated to the nucleus with CSD, further escalating at 24 h. cRel is present in nuclei of a substantial proportion of neurons in the sham group. A further increase in the ratio of neurons with cRel in their nuclei was observed 24 h post-CSD (ANOVA for both cell groups, *P* < 0.001). (**C**) IκB translocation to the nucleus following CSD might contribute to the resolution of the NF-κB-mediated inflammatory response. The significant decrement in the ratio of cells bearing IκB in their nuclei at later time points suggests an acute IκB-mediated regulatory response (Kruskal–Wallis test, *P* = 0.03 for neurons; *P* = 0.002 for non-neuronal cells). Lines and error bars represent the mean and standard error of the mean. Significant pair-wise comparisons (after Bonferroni correction) are denoted by the merging lines on graphs. (**D**) Summary graphs visually depict the change over time in neurons and non-neuronal cells. Constitutive presence of cRel in neuron nuclei is notable. Similar rates of detection for cRel, p65 and IκB in non-neuronal nuclei suggest concomitant activation of pro- and anti-inflammatory transcription. Asterisks indicate the time points significantly differing from the sham surgery group. The centre line of boxes denotes the median value, and the boxes extend from the 25th to the 75th percentile of the dataset. The whiskers mark the 5th and 95th percentiles. CSD = cortical spreading depolarization; IκB = inhibitory kappa B-α; NF-κB = nuclear factor kappa B.

We next evaluated the nuclear translocation (i.e. activation) of NF-κB subunits p65, p50, cRel and IκB in astrocytes 15 min, 5 and 24 h post-CSD (*n* = 3 mice per time point). This investigation was prompted by previous findings demonstrating that pro-inflammatory mediators released from neurons induce NF-κB p65 translocation to astrocyte nuclei, which might initiate inflammatory transcription/signalling.^5,9,13,18^ We first examined nuclear translocation in the cortex ipsilateral to CSD using immunofluorescent labelling. To assess the activation of pro- and anti-inflammatory subunits simultaneously in neurons and astrocytes, we opted for cell phenotyping through immunolabelling with NeuN protein, expressed by all mature neurons in the cortex.^54^ Building upon earlier research from our laboratory, which has consistently shown that p65 translocation is confined primarily to ALDH1L1(+) or S100β(+) astrocytes,^13,18,19,55^ we focused on double staining of subunits with NeuN to differentiate neurons from non-neuronal cells. To confirm this approach, our preliminary double staining with S100β at 24 h reaffirmed that NF-κB cRel nuclear translocation is confined to astrocytes (Supplementary Fig. 2). However, to ensure that microglia, which play pivotal roles in inflammation, were not contributing to the increased NF-κB nuclear immunopositivity in non-neuronal cells at 24 h, we specifically examined co-localization of Iba1 with the three NF-κB subunits. Consistent with our previous observations that NF-κB p65 immunoreactivity remains mostly cytoplasmic (inactive) in microglia 24 h after a single CSD,^19^ in contrast to the response observed with multiple CSDs,^4^ we observed that the other NF-κB members, cRel and p50, were also not translocated to Iba1(+) nuclei 24 h after a single CSD (Fig. 3). Because we also did not observe any nuclear NF-κB translocation in oligodendrocytes, which was identified by their distinctive p65-dominant staining intensity in double immunolabelling studies (see ‘FRET analysis discloses cellular localization of NF-ᴋB subunit pairs’ section and Figs 4 and 5), coupled with their characteristic capping-type cytoplasmic labelling and juxta-neuronal localization,^56^ we concluded that NF-κB nuclear translocation in NeuN(−) non-neuronal cells primarily indicated astrocytic activation. This was substantiated further by the clear distinctions in shape, size and chromatin staining intensity of astrocyte nuclei in comparison to those of microglia and oligodendrocytes.^56^ To ensure an unbiased evaluation, all images were analysed in a blinded manner by the same researcher (Z.K.), without knowledge of the label and the corresponding time point. The immunopositivity of cRel, p50 or p65 in the nuclei (specifically those that were sectioned equatorially and did not contain any cytoplasmic protrusions) was assessed in both NeuN(+) and NeuN(−) cells.

CSD induced p65 activation in non-neuronal cells and, to a much lesser extent, in neurons (Figs 4A and 5A). Relative to the sham surgery group (13% ± 1.5%), the ratio of non-neuronal nuclei exhibiting p65 nuclear translocation increased significantly to 25% ± 2.0% 15 min post-CSD (Tamhane test; *P* < 0.001). In contrast, the proportion of neuronal nuclei displaying positivity was 2% ± 0.6% and 12% ± 1.6%, in sham surgery and post-CSD groups, respectively (Mann–Whitney U-test; *P* < 0.001). Five hours post-CSD, p65 translocation was still significantly high (27% ± 2.5%) in non-neuronal cells, but not 24 h post-CSD (21% ± 2.6%), compared with the sham group (ANOVA; *P* < 0.001).

cRel was activated 15 min post-CSD compared with sham surgery in NeuN(−) cells (24% ± 2.5% and 10% ± 1.3%, respectively; Tamhane test; *P* < 0.001; Figs 4B and 5B). The nuclear translocation of cRel increased further from 5 to 24 h (25% ± 2.0% and 34% ± 2.3%, respectively; ANOVA, *P* = 0.001). These findings suggest that cRel-triggered anti-inflammatory gene transcription might start simultaneously with p65-triggered inflammatory transcription in astrocytes and become even more robust after 24 h, whereas p65 activity decreases. cRel was detected in 32% ± 2.7% of neuronal nuclei after sham surgery. This was unrelated to the sham procedure, because cRel was also present in 33% ± 4.4% of neuronal nuclei in the naïve brain (*n* = 3 mice). This implies that cRel has constitutive transcriptional activity in cortical neurons. No significant changes in cRel activity were observed 15 min and 5 h post-CSD in neurons (25% ± 4.0% and 36% ± 5.5% of all neurons, respectively). However, nuclear cRel increased significantly to 60% ± 3.7% at 24 h, indicating that CSD induces further cRel activation at this time point (ANOVA, *P* = 0.001).

IκB undergoes nuclear translocation following its resynthesis after being degraded as a consequence of pro-inflammatory stimuli. It binds to NF-κB pairs on the DNA, facilitating their transport back to the cytoplasm and rendering them dysfunctional. In order to explore the role of IκB in the resolution of inflammation, we investigated the presence of IκB in the nucleus 15 min, 5 and 24 h post-CSD on brain sections double labelled for IκB and NeuN. For labelling, the IκB-α subtype was preferred owing to its strong association with the classical NF-κB pathway, the activation of which was examined in our study. The ratio of IκB(+) non-neuronal nuclei doubled 15 min post-CSD compared with the sham group (33% ± 4.5% versus 15% ± 2.2%, Mann–Whitney U-test; *P* = 0.002; Figs 4C and 5C). This suggests that translocation of IκB to the nucleus might be one of the steps involved in the resolution of inflammation, in addition to cRel activation in astrocytes. However, the nuclear IκB ratio in non-neuronal nuclei decreased to 27% ± 3.2% and 14% ± 1.4% 5 and 24 h post-CSD, respectively, suggesting that the contribution of IκB to the resolution process might be limited to the acute period. In neurons, the ratio of IκB(+) nuclei was increased 15 min post-CSD in a small population (10% ± 2.1%) but returned to sham levels (4% ± 1.2%) 5 and 24 h post-CSD (3% ± 0.7% and 3% ± 0.9%, respectively; Kruskal–Wallis test; *P* = 0.03). Although the increase at 15 min was not significant compared with sham surgery (*P* = 0.68), this value was significantly higher than ratios at 5 and 24 h (Mann–Whitney U-test; each *P* = 0.011 for two group comparisons). The increase seen in 15 min post-CSD might contribute to suppressing the limited p65 activation in neurons, thus preventing an inflammatory response.

**Figure 6:**
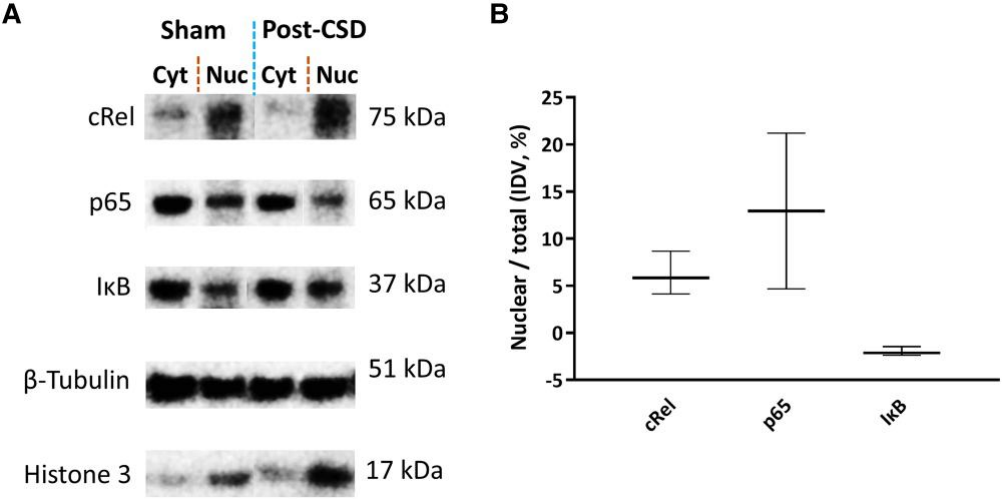
**Verification of pro- and anti-inflammatory NF-κB subunits in the nucleus post-CSD.** (**A**) Western blotting reveals the presence of cRel, p65 and IκB subunits in both the nucleus and the cytoplasm of ipsilateral cortical cells 1 h after sham surgery or CSD. The lanes represent samples from the same blot. (**B**) Although statistically not significant, there is a trend towards increased nuclear fractions of cRel and p65 post-CSD, after subtracting sham values. It is important to note that western blotting can be confounded by tissue extraction process-induced changes (resulting in high activity in the sham group) and a dilution effect of NF-κB subunits from non-neuronal cells, unlike immunofluorescent labelling of *in situ* fixed brain sections. The nuclear fraction of IκB remains indistinguishable between conditions. The centre line denotes the median value, and the upper and lower lines mark the lowest and highest values. CSD = cortical spreading depolarization; IκB = inhibitory kappa B-α; NF-κB = nuclear factor kappa B.

**Figure 7:**
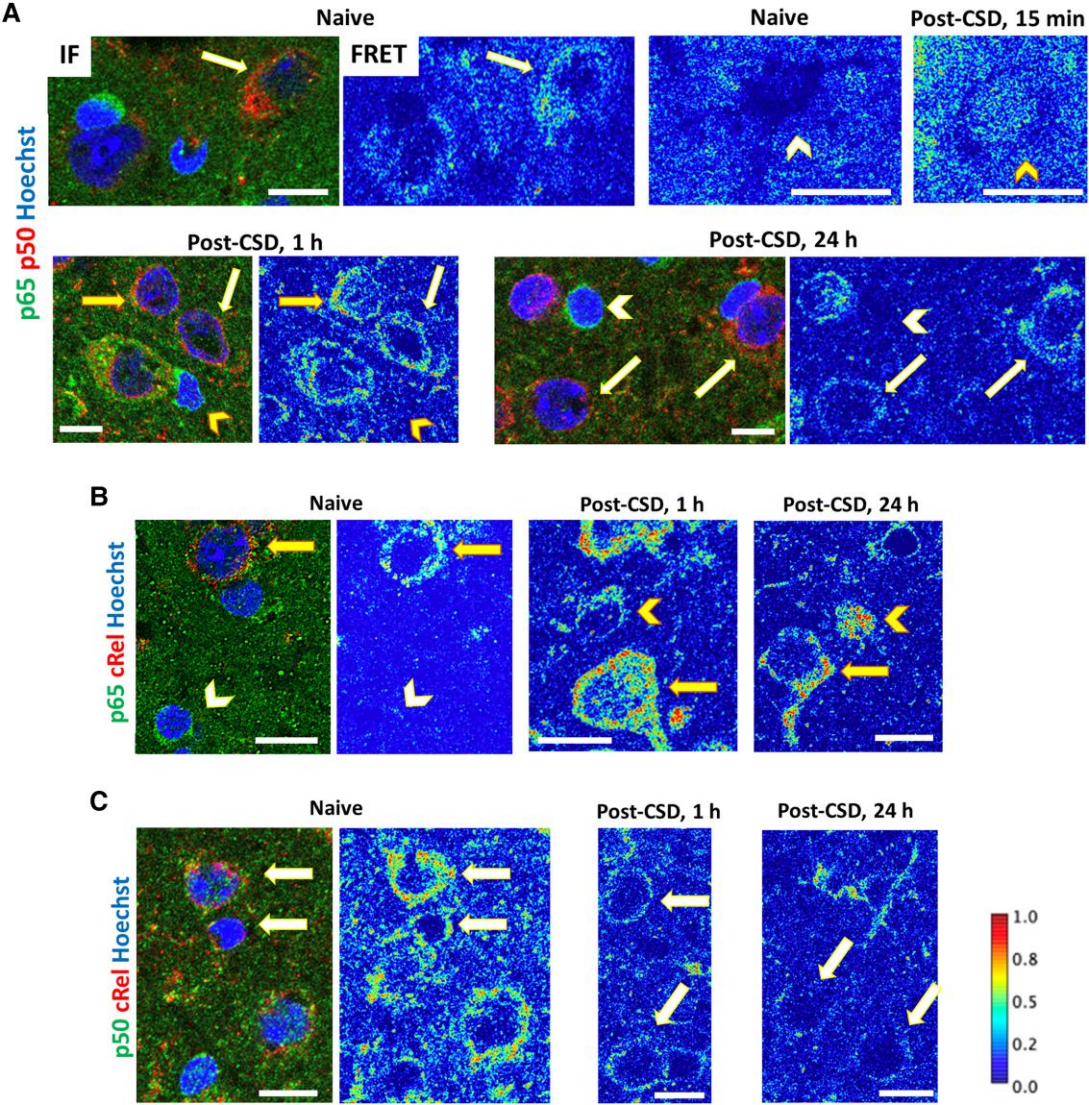
**Evaluation of NF-κB pairs with FRET analysis.** (**A**–**C**) Double IF labelling images and FRET maps for p65:p50, p65:cRel and p50:cRel dimers in representative neurons and astrocytes are presented in **A**–**C**, respectively. The colour codes of IF images are indicated on the left of each panel, while colour scales, maintained equally in the FRET interaction maps, are provided on the right of **C**. Cold colours illustrate low FRET interaction, indicating the absence of the double immunolabelled pair. Morphological criteria and unique double immunolabelling patterns were used to differentiate neurons, astrocytes and oligodendrocytes. Arrows denote neurons without (white arrows) or with (yellow arrows) nuclear FRET pairs. Likewise, yellow arrowheads mark astrocytes with nuclear FRET pairs, and white arrowheads denote astrocytes without nuclear FRET pairs. (**A**) In contrast to the naïve brain, p65:p50 dimers are readily observed in astrocyte and occasional neuron nuclei 15 min and 1 h post-CSD. However, nuclear FRET pairs in both neuron and astrocyte nuclei are not detectable at 24 h post-CSD. (**B**) p65:cRel dimers are present in the nuclei of both astrocytes and neurons at 1 and 24 h post-CSD, with punctate nuclear pairs emerging at 1 h. Notably, p65:cRel dimers are detected in neuronal nuclei in the naïve brain, consistent with constitutional activity highlighted by IF labelling. (**C**) p50:cRel dimers remain cytoplasmic in neurons following CSD. Scale bar: 10 μm. CSD = cortical spreading depolarization; FRET = fluorescence resonance energy transfer; IF = immunofluorescence; NF-κB = nuclear factor kappa B.

A summary graph depicts the temporal distribution of the ratio of cells with cRel(+), p65(+) and IκB(+) nuclei in neurons and non-neuronal cells (Fig. 5D). This simultaneous presentation of the data illustrates parallel changes in the ratios of non-neuronal nuclei over time, suggesting co-localization of these markers in activated astrocytes, despite each label being evaluated on different brain sections owing to technical limitations. It is important to note that co-localization in the same cell does not necessarily imply the formation of pairs, which is the functionally significant aspect. To assess the presence of pairs, we used co-immunoprecipitation on nuclei extracted from brains (Supplementary Fig. 3). Initially, we confirmed the nuclear translocation of NF-κB subunits using western blot analysis of nuclear protein lysates (Fig. 6). Given that co-immunoprecipitation does not provide information about the cell-specific localization of the detected pairs, we also developed an *ex vivo* FRET analysis method to examine cellular distribution of the pairs (Fig. 7).

### Detection of NF-κB subunits in the nucleus by western blotting and co-immunoprecipitation

Owing to the inevitable caspase-1 and downstream NF-κB activation in the period between removing the brain (without fixation) and placing it in the first isolation solution with phosphatase and protease inhibitors, the average of the values obtained after sham surgery was subtracted from the measurements of each sample obtained post-CSD. Examination of nuclear and cytoplasmic proteins separately by western blotting confirmed that both cRel and p65 migrate to the nucleus after CSD (Fig. 6A). The lower increase in nuclear cRel compared with p65 is consistent with the constitutive (baseline) presence of cRel in neuron nuclei (Fig. 6B). Unlike immunofluorescent labelling, western blotting was not sensitive enough to show the additional migration of IκB to a portion of the nuclei induced by CSD over the sham surgery-induced nuclear translocation possibly in a much larger number of cells. Co-immunoprecipitation studies of nuclear lysates verified that subunits translocating to the nucleus formed p65:p50, p65:cRel and p50:cRel pairs (Supplementary Fig. 3).

### FRET analysis discloses cellular localization of NF-κB subunit pairs

We discovered that minimizing donor–acceptor cross-excitation was achievable by using secondary antibodies labelled with AF488 fluorophores as the donor and AF594 fluorophores as the acceptor. This choice was effective because the excitation–emission spectra of this pair were highly suitable for FRET interaction. Consequently, we were able to generate FRET interaction maps at the cellular level on brain sections co-immunostained with antibodies against two NF-κB subtypes. In naïve animals, FRET maps of the cortex revealed that the cytoplasm of neurons contained p65:p50, p65:cRel and p50:cRel pairs, while only p50:cRel pair was distinguishable in the thin astrocyte cytoplasm, probably owing to limited sensitivity in detecting lower-abundance pairs (Fig. 7). Strong FRET interaction signals in cytoplasm of the large neurons and their apical dendrites governed the picture, with the p50:cRel being the most dominant pair in basal conditions. Contrary to the strong and diffuse cytoplasmic signals, nuclear signals were either non-detectable or were visible as scattered puncta of all three heterodimers in neuron nuclei. No pairs could be detected in astrocyte nuclei in naïve mice, except for occasional p65:p50 puncta. However, 15 min to 1 h post-CSD, both p65:p50 and p65:cRel but not p50:cRel pairs were detected in astrocyte nuclei and in the cytoplasm, where FRET signals were not detectable in basal conditions. Nuclear p65:p50 pairs disappeared while p65:cRel signal continued to be observed 24 h post-CSD in both astrocyte and neuron nuclei.

### RNA sequencing shows cell-specific activation of inflammatory and anti-inflammatory genes

For cell-specific RNA sequencing, neuron and non-neuronal cells were isolated from the ipsilateral hemicortex. Owing to the activation of inflammatory mechanisms during brain extraction and processing, as observed in the above experiments without *in vivo* fixation, cell dissociation and one-step separation (neuronal versus non-neuronal) were performed rapidly and gently using the magnetic-activated cell sorting technology as quickly as possible. Unanaesthetized Thy1-ChR2-YFP mice were used for the transcriptomic study to avoid the well-known effects of anaesthesia and surgical manipulations on gene transcription. Cortices were obtained from transgenic mice at 1 h post-optogenetic CSD and from sham-operated controls (*n* = 5 per group). We selected this time point based on a study examining gene expression following a 5 min depolarization induced by KCl or bicuculline, which found that transcript levels peaked at the 1 h time point.^57^ From each group, three of five samples having the highest RNA integrity number score and RNA concentration were selected for sequencing. The transcriptome profile of the neurons and non-neuronal cells isolated after CSD or sham surgery was determined by RNA sequencing of the 12 selected samples. Following confirmation of the quality of the sequencing data, differentially expressed genes between the groups were identified. The substantial and significant differences in the distribution of cell-specific ‘marker’ genes in both CSD and sham groups confirmed the effective separation of neurons and non-neuronal cell populations (Supplementary Table 2).

Subsequently, we investigated inflammation-related pathways specifically by performing the KEGG pathway analysis in both cell populations. Remarkably, inflammatory pathway genes did not exhibit significant changes in neurons 1 h post-CSD (Supplementary Table 5). In contrast, non-neuronal cells exhibited a significant difference in several pathways, including ‘cytokine–cytokine receptor interaction’, ‘TNF signalling’, ‘NF-κB signalling’, ‘chemokine signalling’ and ‘C-type lectin receptor signalling’ as revealed by KEGG pathway analysis (Supplementary Table 5). Despite the significant *P*-values, the non-significant q-values observed in some pathways are likely to stem from the confounding effect of procedure-triggered inflammatory transcription. This effect tends to diminish the differences between the CSD and sham groups and increase variability. Additionally, the non-uniform behaviour of these pathways in astrocytes and microglia might contribute further to this phenomenon, as clarified below.

To gain insight into the contribution of astrocytes and microglia to the inflammatory transcriptional response, we used Bayesian cell proportion reconstruction inferred using statistical marginalization (BayesPrism),^58^ a tool for predicting the cell type composition and gene expression profile of each cell type in bulk RNA sequencing using a reference single-cell RNA-sequencing dataset. As a reference,^59^ we used single-cell transcriptomes from cortical regions of mouse brains and obtained astrocyte- and microglia-related gene sets that were separated from the non-neuronal RNA pool using BayesPrism. The results showed a significant difference in the distribution of astrocyte- and microglia-specific cell-marker transcripts in both CSD and sham groups, confirming the effective separation of astrocyte and microglia populations (Supplementary Table 6). Additionally, this analysis unveiled distinct patterns in the activation of inflammatory signalling molecules in astrocytes and microglia in response to CSD.

Consistent with the co-translocation of pro- and anti-inflammatory NF-κB dimers to the astrocyte nucleus, BayesPrism analysis revealed that 1 h after a single optogenetically induced CSD, astrocytes exhibited induction of both protective/anti-inflammatory and inflammatory transcripts (Table 1). This dual response suggests a tightly regulated inflammatory process from the outset, potentially aimed at preventing excessive inflammation and astrogliosis while allowing for controlled inflammatory signalling (Table 1 and Supplementary Table 7). Specifically, we observed significant induction of NF-κB-targeted survival genes, accompanied by downregulation of pro-inflammatory transcripts in astrocytes, although not all adjusted *P*-values reached significance, which might be attributable to a heterogeneous response among cortical astrocytes. Additionally, NF-κB-unrelated inflammatory transcripts, such as S100 calcium-binding protein A1 (*S100a1*) and bradykinin receptor B2 (*Bdkrb2*), were also upregulated (Table 1 and Supplementary Table 7). Likewise, in microglia, numerous mediators showed up- or downregulated expression, with many trending towards an anti-inflammatory profile (e.g. downregulation of *Tlr4*, *Tlr7* and *Il7r* or upregulation of *Bcl2a1* transcripts; Table 1 and Supplementary Table 8). However, a contrasting pattern emerged with the upregulation of several chemokines and cytokines, including tumour necrosis factor (TNF) and TNF-induced proteins (Table 1 and Supplementary Table 8), suggesting that microglia might play a role in sustaining inflammatory/anti-inflammatory responses in astrocytes during subsequent hours through the release of these mediators.^38^ Brief exposure to ATP during CSD might activate TNF release via P2X7 receptors and the p38 MAP kinase pathway, as observed in cultured microglia.^60,61^ ATP has been reported to increase TNF mRNA levels in microglia at the post-transcriptional level by stabilizing TNF mRNA and facilitating its nucleocytoplasmic transport.^60,61^ The upregulation of genes involved in chemotaxis, such as *Ccl3* (Table 1 and Supplementary Table 8), suggests that microglial processes might be activated to extend towards CSD-injured synapses, as previously reported.^62-66^ Supporting this, genes encoding the C-chain polypeptide of complement subcomponent C1q (a key mediator of microglia-mediated spine pruning) demonstrated a subtle yet highly significant upregulation of 15% (*P* = 0.0005, adjusted *P* = 0.0492) in microglia.^67-69^

**Table 1:** Differentially expressed inflammation-related genes in astrocytes and microglia with their potential impact

Astrocytes		Microglia	
Potential impact of transcription change		Potential impact of transcription change	
Anti-inflammatory/protective	Inflammatory	Anti- inflammatory/protective	Inflammatory
*Agt* ↑	*Bdkrb2* ↑	*Bcl2a1d* ↑	*Tnfaip2* ↑
*Nr4a3* ↑	*Itih3* ↑	*Bcl2a1b* ↑	*Ccl3* ↑
*Apod* ↑	*S100a1* ↑	*Nr4a3* ↑	*Tnf* ↑
*Mt3* ↑	*Ptpn13* ↓	*Cd83* ↑	*Ccl9* ↑
*Csf1* ↑	–	*Gadd45b* ↑	*Nr4a1* ↑
*Tnc* ↓	–	*Apod* ↑	*Thbs1* ↓
*Hgf* ↓	–	*Cd68* ↑	*Cd163* ↓
–	–	*Csf1* ↑	–
–	–	*Ccrl2* ↑	–
–	–	*Ccr1* ↓	–
–	–	*Il7r* ↓	–
–	–	*Tlr4* ↓	–
–	–	*Ccr5* ↓	–

The direction of transcriptional changes is indicated by arrows. Transcripts are listed from top to bottom, with the highest magnitude of change at the top. Detailed information is provided in Supplementary Tables 6 and 7.

## Discussion

We found that the pro-inflammatory stimuli from neurons that drive inflammatory signalling in astrocytes/microglia diminished a few hours after a single CSD. After the initial burst, there was no more HMGB1 release from neurons, and the caspase-1 activation faded within 5 h. Notably, pro-inflammatory NF-κB p65:p50 pairs, alongside anti-inflammatory cRel:p65 pairs, were detected in astrocyte nuclei shortly after CSD. Interestingly, 24 h post-CSD, the nuclear p65:p50 pairs disappeared while cRel:p65 persisted, indicating a shift from pro-inflammatory dominant to anti-inflammatory transcriptional activity in astrocytes. Cell-specific transcriptomic data confirmed NF-κB-related pro- and anti-inflammatory transcription in astrocytes 1 h post-CSD, whereas no such activity was observed in neurons. The inflammatory molecules not regulated by NF-κB, such as *Bdkrb2*, which mediates prostaglandin release,^70^ were also upregulated in astrocytes. Cell-specific analysis also revealed that microglia exhibited a transcriptional anti-inflammatory profile in addition to the upregulation of several chemokines and cytokines, including TNF. These segregated transcriptional changes in neurons, astrocytes and microglia might facilitate optimal post-CSD tissue restoration and repair while reporting stress through tightly controlled inflammatory signalling in astrocytes. This signalling could potentially influence or modulate the meningeal nociceptor activity through an extensive astrocyte endfeet syncytium abutting subarachnoid and perivascular spaces.^26,27^ Supporting this, a recent study proposed that inflammatory mediators released from astrocytes could diffuse and directly activate the trigeminal ganglion through openings in the meninges.^28^ This nuanced understanding of the inflammatory response across different cell types highlights the complex cellular interactions and responses to CSD.

The observed absence of HMGB1 release from neurons after the initial burst and the decrease in neuronal caspase-1 activity within 5 h are anticipated to reduce the inflammatory stimulus in astrocytes. The enhanced caspase-1 p20 immunolabelling and the shift from punctate to diffuse staining 3 h post-CSD are hypothesized to result from the dispersal of p20 monomers separated from p20/p10 tetramers on the inflammasome complex to the cytoplasm.^49^ Although free cytoplasmic p20 is more accessible to antibodies, yielding higher-intensity labelling, it also signals the end of caspase-1 activity, because free p20 lacks enzymatic activity and is destined to be degraded into smaller fragments.^49^ In another study conducted in our laboratory, the increased ratio of neurons with cytoplasmic IL-1β (the product of caspase-1 activity) immunopositivity returned to basal values 6 h after multiple CSDs.^71^ This provides further support for the notion that the observed changes in caspase-1 activity might represent a transient response and suggest a regulatory mechanism in the resolution of the inflammatory signalling cascade.

We showed that p65 migrated to the astrocyte nuclei shortly after CSD together with cRel. However, the observed decrease in p65 activation within 24 h, coupled with an increase in cRel nuclear positivity, suggests a nuanced regulation wherein both pro- and anti-inflammatory gene expression are activated concomitantly, but the balance shifts towards the anti-inflammatory side over time. It is crucial to note that the co-localization in immunofluorescent labelling of two subunits in the same nucleus does not necessarily imply the formation of a pair and the induction of transcription. For instance, p65 can form both the anti-inflammatory cRel:p65 pair and the pro-inflammatory p65:p50 pair in the nucleus.^72,73^ While the p65:p50 promotes the expression of inflammatory genes, the cRel-containing pairs induce the expression of anti-inflammatory/survival genes.^35-37,39-41,74^ The relative abundance of these pairs and their binding to DNA competitively, along with the co-presence of other transcriptional regulators, determines the overall behaviour of the nucleus.^75-77^ Furthermore, NF-κB pairs can influence the transcription of various NF-κB subunits and IκB proteins, leading to a dynamic and changing dominant action within the same cell over time. For example, in a study conducted in macrophages, it was shown that activation of the p65:p50 pair after lipopolysaccharide stimulation increased cRel expression, and the cRel:p65 pairs formed subsequently mediated resolution of the lipopolysaccharide-induced inflammation.^78^ One of the steps that terminate NF-κB activation is IκB, which passes into the cell nucleus, binds to NF-κB pairs in the nucleus and renders them dysfunctional. Consistent with this, the presence of IκB in astrocyte nuclei along with p65 and cRel shortly after CSD, suggests a potential role in terminating p65:p50 activity. However, these possibilities and their time course need to be investigated in future studies to gain a better understanding of the specific mechanisms involved in the termination of NF-κB activation after CSD.

Our comprehensive analyses, including conventional co-immunoprecipitation and FRET, shed light on the dynamic and cell-specific aspects of NF-κB pair formation in response to CSD. The finding that both p65:p50 and p65:cRel pairs were present in astrocyte nuclei and cytoplasm within 1 h post-CSD, with the disappearance of nuclear p65:p50 pairs and continued observation of p65:cRel signals 24 h post-CSD in astrocytes, suggests a shift towards anti-inflammatory transcription by the later time point. In neurons, the initial pro-inflammatory trend might have been balanced by the constitutive presence of cRel, followed by a substantial increase in p65:cRel pairs, indicating tight control over pro-inflammatory transcription in neuronal nuclei.^35,37^ This aligns with the transcriptomic data showing the absence of activation of inflammatory pathways in neurons. The transcriptional upregulation of NF-κB, toll-like receptor and advanced glycation end products (AGE)/receptor for AGE (RAGE) signalling pathways in non-neuronal cells after CSD supports the notion of NF-κB-mediated pro-inflammatory action through HMGB1 and toll-like and/or RAGE receptors.^7^ The absence of detectable NF-κB translocation to microglial nuclei, in contrast to astrocytes, indicates a distinct role for microglia in response to a single CSD. Notably, several upregulated survival or pro-inflammatory transcripts are NF-κB targets, suggesting either rapid post-translational modification of constitutively expressed NF-κB transcripts (e.g. by stabilizing their mRNAs) in microglia or the involvement of other transcriptional regulators (e.g. TNF) binding to regulatory regions of these genes.^60,61^ Alternatively, some NF-κB transcripts detected might have originated from perivascular macrophages, because the reference transcriptomic dataset cannot distinguish between these two cell types.^38,59^ The release of HMGB1 within small extracellular vesicles from the soma, acting preferentially on astrocyte processes extensively covering neuronal soma rather than dynamic microglia contacts, is consistent with the absence of histologically detectable NF-κB translocation to the nucleus in microglia and provides additional insights into the distinct responses of microglia to CSD.^19^ The downregulation of *Tlr4* and *Tlr7* transcription in microglia might additionally contribute to the differential gene expression patterns in microglia compared with astrocytes. Overall, our findings suggest that astrocytic inflammatory signalling plays a role in reporting neuronal stress, as shown before,^18^ whereas microglial signalling activated by microglia–synapse crosstalk, a well-documented phenomenon both in physiological conditions and following injury,^62-66^ might be involved in regulating repair processes at swollen spines during CSD.^79^ Future studies will be likely to provide more details on the cell-specific inflammatory transcriptomic changes and the intricate interplay between different cell types in response to CSD.

Several prior studies have explored transcriptomic changes following CSD by measuring bulk mRNA levels in brain extracts.^6,9,80-82^ Although these investigations also noted an upregulation in the expression of inflammation-related genes, such as *Tnfa*, *Il1β* and *Cox2*, in the cortex, they did not delve into the cellular origin of these transcriptomic changes. Takizawa *et al*.^9^ found that the transcription levels of *Cox2* and *Il1β* increased, whereas *Il-6* remained unchanged in the ipsilateral cortex compared with the contralateral side following optogenetically induced single CSD. This differs from the changes observed in studies of multiple CSDs induced by KCl, highlighting that the transcriptional response varies between single and multiple CSDs. This is notable because NF-κB is translocated to the nuclei of microglia 24 h after multiple CSDs, in contrast to our findings. We opted for a single CSD model because it better mirrors migraine with aura attacks, which are typically preceded by a single aura (i.e. CSD).^83,84^ This choice is likely to allow us to capture a transcriptomic profile that is more reflective of the physiological conditions associated with migraine attacks. Given the well-documented upregulation of numerous inflammatory transcripts, we focused our analysis on the 1 h time point following CSD to examine early transcriptional changes in a cell-specific manner before reciprocal interactions could potentially complicate the transcriptomic landscape. Additionally, our unbiased transcriptomic profiling facilitated the detection of anti-inflammatory transcriptional activity and the identification of several previously unrecognized inflammatory and anti-inflammatory transcripts. Altogether, the transcriptomic findings underscore the highly complex nature of the inflammatory response to CSD and highlight the need for further studies at the protein and functional levels to deepen our understanding of these therapeutically important mechanisms in headache generation and termination.

## Conclusion

In summary, a single CSD elicits distinct transcriptomic responses in neurons, astrocytes and microglia. Neurons benefit from protection against inflammatory responses through cRel, while astrocytes undergo a strictly controlled transient pro-inflammatory signalling phase coupled with anti-inflammatory transcriptional activity. Microglia display a unique expression phenotype, which might possibly be associated with synaptic repair and sustaining astrocytic inflammatory activity. The intricate transcriptional cascade is initiated by neurons releasing pro-inflammatory mediators, such as HMGB1 and IL-1β. The release of HMGB1 is transient, and caspase-1 activity (i.e. IL-1β release) diminishes within 5 h. These data suggest that inflammatory signalling induced by a single CSD is tightly regulated and largely resolved within 24 h. We hypothesize that the resolution of parenchymal inflammatory transcriptional activity might be a prerequisite for suppressing dural neurogenic inflammation and terminating headache.

## Supplementary Material

awaf015_Supplementary_Data

## Data Availability

The data that support the findings of this study are available from the corresponding author, upon reasonable request.
