# Supplementary material for: Spreading depolarization triggers pro- and anti-inflammatory signalling: a potential link to headache
Source: Brain. 2025 Jan 17;148(7):2522–36. doi: 10.1093/brain/awaf015 (PMC12378585; doi:10.1093/brain/awaf015)
Supplement: awaf015_Supplementary_Data [file awaf015_Supplementary_Data.pdf]

Supplementary Methods

Induction of CSD

The experiments were carried out according to the Guide to the Care and Use of Laboratory Animals and reported per the ARRIVE guidelines. The animals were housed under a 12-hour light-12-hour dark cycle at a temperature of 22 ± 3°C and 40–60% humidity and allowed free access to food and water. Both strains were bred by the Hacettepe University Experimental Animal Facility.

Swiss mice were anesthetized with xylazine (10 mg/kg, intraperitoneal (ip)) and urethane (1.25 g/kg, ip, U2500, Sigma-Aldrich) or isoflurane (1.5-2%) under continuous oxygen delivery (2 l/min) and were placed in a stereotaxic frame (Digital Lab Standard Stereotaxic Frame, Stoelting). Body temperature was monitored with a rectal probe and maintained at 37.0±0.2°C by a homeothermic blanket control unit (Kent Scientific). Pulse rate and oxygen saturation were monitored by an oximeter using a mini Y-clip hind paw probe (The LifeSense® VET pulse oximeter, Nonin Medical Inc.). The parietal bone was thinned using a microdrill (Fine Science Tools, USA), and a 1.5-mm burr hole was opened over the frontal region of the right hemisphere (1 mm anterior and 1 mm lateral to bregma). The skull was irrigated with cold saline to prevent complications due to heating caused by the drilling procedure. The dura under the burr hole was kept intact and maintained moist by repeated applications of aCSF preheated to 37°C until the experiment started. An Ag-AgCl pellet electrode was placed over the thinned parietal bone to record the direct current (DC) potential changes. EEG gel was applied to the electrode tip to enhance electrical contact with bone. A reference electrode was placed between the layers of the neck muscles. DC potential changes, heart rate, and tissue oxygen saturation were recorded using the Lab Chart data acquisition system (AD Instruments). A single CSD was induced by pinpricking the cortex and verified with the DC potential shift observed.

In order to trigger CSD via optogenetics in an unanesthetized animal, an opto-electric cannula (optrode) was designed. The optical fiber was 400 µm in diameter with a numerical aperture of 0.48. A brass ring that we produced fixed the optrode to the skull with a mixture of cyanoacrylate and dental acrylic. Since this system was connected to the recording device with the help of a light and mobile cable and a swivel system, it did not create a significant weight on the animal's head, allowing comfortable movement and electrophysiological recording of acceptable quality from the moving animal. In order to further reduce the possible discomfort, the mice were allowed to get used to the device by being tied to the device for 30 minutes, twice

a day, for 2 days before the experiment. A 450-nm light stimulation at a power of approximately 1mW was applied uninterruptedly for 10 seconds to trigger CSD. This stimulation protocol has previously been optimized for our laboratory and reliably results in a CSD for every application.

Immunofluorescent Labeling

Mice were deeply anesthetized and transcardially perfused with 0.4% heparinized saline and 4% paraformaldehyde (PFA). The brains were quickly removed, postfixed in the same PFA solution overnight, and cryoprotected in 30% sucrose solution for two days. Thirty-five micron-thick coronal sections were cut on a sliding microtome (for activated caspase-1 labeling, SM2000 R, Leica) and 8 or 20 micron-thick coronal sections were cut on a freezing cryostat (CM1100, Leica GmbH). Sections were blocked with either 10% normal goat serum, 10% normal donkey serum, or 3% bovine serum albumin in phosphate-buffered saline (PBS) according to the species in which the secondary antibody is produced. The sections were mounted in glycerol/PBS (1:1) medium containing 12.5 mg/ml sodium azide and 1 μl/ml Hoechst-33258 (H3569, Thermo Fisher Scientific) for nuclear identification. Primary antibody omission incubations with blocking solution were performed to test the specificity of immunoreactivity.

Western Blotting

Samples and albumin standards were prepared as instructed by the manufacturer, and their absorbance at 562 nm was measured with a spectrophotometer (Infinite F50, Tecan). Equal amounts of protein were loaded on 12% SDS–PAGE gels and subsequently transferred to PVDF membranes by semidry transfer. Nonspecific protein binding was blocked by incubating the PVDF membranes in 5% skim milk powder for an hour, and then the membranes were incubated with antibodies against caspase-1 (1:500), cRel (1:3000), p65 (1:3000), histone 3 (1:1000, 9715, Cell Signaling Technology), overnight at +4°C. The next day, after washing, the membrane was incubated with horseradish peroxidase-conjugated goat anti-mouse or anti-rabbit IgG secondary antibody (1:5000, ab6789 or ab6721, respectively, Abcam) for 1 or 2.5 hours at RT. The protein bands were visualized with the chemiluminescence method (34094, Super Signal West-Femto, Thermo Fisher Scientific), and the images were captured by Image Station 4000 (Kodak). After the membrane was stripped off the antibody complexes, it was incubated with either β-actin (1:5000, ab8226, Abcam) or β-tubulin (1:5000, ab6046, Abcam) for 1-hour at RT and reprobed with the appropriate secondary antibody as mentioned before. ImageJ (National Institute of Health, USA) was used for densitometric analysis.

To analyze activated caspase-1 by Western blotting, the integrated intensity value (IDV) of the pro-caspase-1 (45 kDa) and cleaved (active) caspase-1 (20 kDa) were measured. The fraction of activated enzyme was calculated by comparing IDVs of the active caspase-1 band to total enzyme (active + pro form). β-actin was used as a loading control to compare the total enzyme amount.

Co-immunoprecipitation

A commercial kit was used for the co-immunoprecipitation (Pierce Co-Immunoprecipitation Kit, 26149, Thermo Fisher Scientific) following the manufacturer's instructions with minor optimizations. Anti-p65 and anti-cRel antibodies (8242S and 4727S, respectively, Cell Signaling Technology) were conjugated overnight with coupling resin having amine reactivity. The antibody-conjugated beads were then incubated with 250 µg of nuclear protein lysate for 24 hours. After repeated washing, a buffer with low pH (Glycine-HCl; pH 2.05) was used for the elution of the protein pairs, and the antibody-bead complexes were precipitated by centrifugation. The protein concentration of the supernatant was determined by the BCA assay (23227, Thermo Fisher Scientific). After capturing either p65 or cRel-containing pairs, the other subunit of pair was detected using Western blotting. For this, anti-p65 (1:1000), anti-p50 (1:500, sc-166588, Santa Cruz Biotechnology), and anti-cRel (1:1000, sc-6955, Santa Cruz Biotechnology) antibodies were incubated with membranes at +4°C overnight. After washing, the membrane was incubated with horseradish peroxidase-conjugated secondary antibody (1:2500, ab6789 or ab6721, Abcam) for 3 hours at RT.

Tissue Dissociation and Isolation of Neurons by Magnetic-Activated Cell Sorting (MACS)

In brief, the mouse brain cortex and subcortical structures were separated on ice using a round-edged spatula under a microscope, minimizing subcortical tissue contamination. The tissue was cut into 1 mm^3 pieces and placed in the C-tube containing 1950 µl of enzyme mix 1 (Enzyme P and Buffer Z). Mechanical and enzymatic tissue dissociation was performed using the gentleMACS Dissociator (130-093-235, Miltenyi Biotec) with the "m_brain_01" program. The sample was incubated with rotation for 15 minutes at 37 °C. Subsequently, 30 µl of enzyme mix 2 (Enzyme A and Buffer Y) was added to the C-tube, and the "m_brain_02" program was applied. Following another incubation step with rotation for 10 minutes at 37 °C, the C-tube was placed onto the gentleMACS Dissociator, and "m_brain_03" was run. Incubation with rotation for an additional 10 minutes at 37 °C was then applied. Following digestion, samples

were strained through a 70-µm filter and pelleted by centrifugation. Cell debris and red blood cell removal steps were performed according to the manufacturer's instructions.

In this protocol, non-neuronal cells labeled with biotin-conjugated monoclonal antibodies (an antibody mixture specific to astrocytes (anti-ACSA-2-PE), oligodendrocytes (anti-O4-PE), microglia (anti-CD11b-FITC), and endothelial cells (anti-CD31-PE)) were depleted by retaining them within a MACS® Column in the magnetic field of a MACS Separator with the help of anti-biotin MicroBeads. Briefly, approximately 10^7 cells were resuspended in 80 µl of PBS/BSA (PB) buffer and incubated for 5 minutes at 4°C with 20 µl of the non-neuronal cell biotin-antibody cocktail, followed by washing with 1 ml of PB buffer and centrifugation at 300 g for 10 minutes. Cells were then resuspended in 500 µl of PB buffer and applied onto a MACS LS Column fitted into a MACS Separator (130-042-401, 130-042-302, respectively, Miltenyi Biotec). Unlabeled neuronal cells were collected with two 1 mL washes. Labeled non-neuronal cells remained captured in the magnetic field. The column was removed from the magnetic separator, and non-neuronal cells were eluted by firmly pressing the plunger into the column.

RNA Isolation

Briefly, cells were incubated with 500 µl of TRIzol for 5-10 minutes at RT. After incubation with 100 µl of chloroform for 10-15 minutes at RT, the solution was centrifuged at 12,000 g at 4ºC for 15 minutes and the supernatant was transferred to a clean tube. Following the addition of 500 µl of isopropanol, the sample was centrifuged at 12,000 g at 4ºC for 15 minutes and the supernatant was discarded. Subsequently, 500 µl of 75% ethyl alcohol was added to the pellet and centrifuged at 7,500 g for 10 minutes at 4ºC; then the supernatant was removed. After the alcohol evaporated, the RNA was dissolved in RNAse-free water.

Supplementary Table 1. Immunofluorescent labeling protocols in detail.

Labeling	Primary antibodies and incubation (at +4°C)	Secondary antibodies and incubation (at RT)	Antigen retrieval technique
Activated caspase-1	* Invitrogen, PA5-38099, 1:100<br>* 48 h, free-floating sections	* Jackson ImmunoResearch (JIR), 111-225-144, 1:200<br>* 2 h, RT, free floating	None
HMGB1	* Abcam, ab18256, 1:200<br>* overnight (ON)	* JIR, 111-225-144, 1:200<br>* 90 min	None
Iba1-p65	* Novus Biologicals (NB), NB100-1028, 1:200<br>* Cell Signaling Technology (CST), 8242, 1:100<br>* together, ON	* JIR, 705-165-147, 1:200<br>* Abcam, ab96891, 1:200<br>* together, 1 h	Sodium citrate buffer (pH=6), 80 °C, 15 min
Iba1-cRel	* NB, NB100-1028, 1:200<br>* Santa Cruz (SC), sc-6955, 1:200<br>* together, ON	* JIR, 705-165-147, 1:200<br>* Abcam, ab96875, 1:200<br>* together, 1 h	Sodium citrate buffer (pH=6), 80 °C, 15 min
Iba1-p50	* NB, NB100-1028, 1:200<br>* SC, sc-166588, 1:200<br>* together, ON	* JIR, 705-165-147, 1:200<br>* Abcam, ab96875, 1:200<br>* together, 1 h	Sodium citrate buffer (pH=6), 80 °C, 15 min
NeuN-p65	* Chemicon, MAB377B, 1:200<br>* CST, 8242, 1:200<br>* together, 24 h	* JIR, 115-165-146, 1:200<br>* JIR, 111-225-144, 1:200<br>* together, 90 min	Triton X-100 (0.2%), RT, 10 min
NeuN-cRel	* CST, 24307, 1:200<br>* SC, sc-6955, 1:200<br>* consecutively, 24 h	* JIR, 111-165-144, 1:200<br>* JIR, 115-225-146, 1:200<br>* consecutively, 90 mi	None
NeuN-IκB	* CST, 24307, 1:200<br>* CST, 4814, 1:200<br>* consecutively, 24 h	* JIR, 111-165-144, 1:200<br>* JIR, 115-225-146, 1:200<br>* consecutively, 90 min	None
p65-p50	* CST, 8242, 1:100<br>* SC, sc-166588, 1:200<br>* together, ON	* Abcam, ab150116, 1:200<br>* Abcam, ab150077, 1:200<br>* consecutively, 1	Sodium citrate buffer (pH=6), 80 °C, 15 min
p65-cRel	* CST, 8242, 1:100<br>* SC, sc-6955, 1:200<br>* together, ON	* Abcam, ab150116, 1:200<br>* Abcam, ab150077, 1:200<br>* consecutively, 1	Sodium citrate buffer (pH=6), 80 °C, 15 min
p50-cRel	* SC, sc-166588, 1:200<br>* CST, 4727S, 1:200<br>* together, ON	* Abcam, ab150116, 1:200<br>* Abcam, ab150077, 1:200<br>* consecutively, 1 h	Sodium citrate buffer (pH=6), 80 °C, 15 min

Supplementary Table 2. Cell-specific transcripts verify successful separation of neurons and nonneuronal cells for CSD and sham surgery groups. log2 Fold Change>0 denotes neurons whereas log2 Fold Change <0 denotes nonneuronal cells.

	**CSD**		**Sham Surgery**	
	**log2 Fold Change**	**q value**	**log2 Fold Change**	**q value**
Neuron-specific transcripts				
Rbfox3	3.074	**1.9E-07**	1.695	**1.46E-03**
Syp	1.832	**4.2E-05**	1.257	**1.15E-02**
Nefh	3.055	**7.99E-03**	3.143	**4.52E-04**
Astrocyte-specific transcripts				
Aldh1l1	-4.657	**8.55E-35**	-7.394	**2.49E-06**
Gfap	-3.266	**1.14E-03**	-3.524	**2.21E-05**
S100b	-4.192	**1.81E-37**	-4.237	**1.47E-04**
Slc1a2	-4.938	**1.28E-40**	-5.950	**4.50E-16**
Oligodendrocyte-specific transcripts				
Mbp	-5.196	**1.48E-43**	-4.927	**8.71E-08**
Mog	-4.773	**9.07E-82**	-3.297	**9.98E-03**
Olig1	-5.725	**1.17E-14**	-4.613	**3.22E-13**
Olig2	-5.449	**5.65E-20**	-6.235	**1.84E-08**
Microglia-specific transcripts				
Tmem119	-3.242	**2.73E-36**	-2.159	**2.37E-02**
Itgam	-5.246	**2.2E-99**	-4.885	**9.80E-06**

Supplementary Table 3. Comparison of the three brains in each group showing that the groups were homogeneous as indicated by Pearson correlation coefficients.

**Samples**	**Neurons**	**Nonneuronal cells**
CSD1 vs CSD2	0.862	0.973
CSD2 vs CSD3	0.942	0.922
CSD1 vs CSD3	0.930	0.920
Sham1 vs Sham2	0.457	0.911
Sham2 vs Sham3	0.681	0.878
Sham1 vs Sham3	0.806	0.961

Supplementary Table 4. HMGB1 is replaced within 72 hours post-CSD.

	**HMGB1 (+) nuclei (% of all, mean±SE)**	**HMGB1 (-) nuclei (% of naive, mean±SE)**
Naive	83±1.8	-
15 minutes post-CSD	63±2.5	23±3.0
24 hours post-CSD	61±3.3	26±1.8
72 hours post-CSD	81±3.4	2±1.8

Supplementary Table 5. KEGG inflammation-related pathways in neurons and nonneuronal cells.

	Term Candidate Gene Num	Total Candidate Gene Num	Term Gene Num	Total Gene Num	Rich Ratio	p value	q value
**Nonneuronal cells**							
Cytokine-cytokine receptor interaction	18	174	319	8650	0.05643	**0.00007**	**0.00248**
TNF signaling pathway	8	174	141	8650	0.05674	**0.00749**	0.16374
NF-κB signaling pathway	7	174	122	8650	0.05738	**0.01141**	0.22188
Chemokine signaling pathway	9	174	216	8650	0.04167	**0.03017**	0.31059
C-type lectin receptor signaling pathway	6	174	122	8650	0.04918	**0.03625**	0.33394
**Neurons**							
Cytokine-cytokine receptor interaction	14	426	295	8491	0.04746	0.62514	0.86793
TNF signaling pathway	7	426	113	8491	0.06195	0.33892	0.58588
NF-κB signaling pathway	5	426	108	8491	0.04623	0.63689	0.87257
Chemokine signaling pathway	16	426	197	8491	0.08122	**0.03873**	0.18953
C-type lectin receptor signaling pathway	9	426	112	8491	0.08036	0.10908	0.32937

Supplementary Table 6. Astrocyte and microglia-specific transcripts verify effective separation of astrocyte and microglia populations for CSD and sham groups using Bayesprism. log2 Fold Change>0 denotes astrocytes whereas log2 Fold Change< 0 denotes microglia.

	**CSD**			**Sham Surgery**		
	**log2 Fold Change**	**p value**	**q value**	**log2 Fold Change**	**p value**	**q value**
Astrocyte-specific transcripts						
Aldh1l1	6.043	**1.5E-220**	**1.8E-218**	6.036	**2.76E-195**	**5.4E-193**
Gfap	9.681	**9.87E-39**	**1.119E-37**	9.364	**1.441E-36**	**1.91E-35**
S100b	4.756	**3.81E-85**	**1.018E-83**	4.727	**2.047E-10**	**9.66E-10**
Slc1a2	6.817	**0**	**0**	6.815	**0**	**0**
Slc1a3	4.207	**0**	**0**	4.205	**1.16E-197**	**2.34E-195**
Microglia-specific transcripts						
Tmem119	-10.693	**5.29E-71**	**1.12E-69**	-10.648	**5.684E-98**	**2.77E-96**
Cd68	-7.401	**2.4E-175**	**1.86E-173**	-7.345	**2.25E-153**	**2.34E-151**
Ptprc	-9.946	**3.06E-32**	**3.029E-31**	-9.921	**2.475E-50**	**4.69E-49**

Supplementary Table 7. Upregulated and downregulated inflammation-related genes in astrocytes.

**Gene**	**Encoded protein**	**log2 Fold Change**	**p value**	**Adjusted p value**	**Fold change (%)**	**Potential impact of transcription change**
**Upregulated genes**						
**Agt**	Angiotensinogen	1.35	**0.000**	**0.000**	182%	anti-inflammatory
**Bdkrb2**	Bradykinin receptor B2	1.21	**0.005**	1.000	147%	inflammatory
**Itih3**	Inter-alpha-trypsin inhibitor heavy chain 3	0.92	**0.000**	**0.002**	85%	inflammatory
**Nr4a3**	Nuclear receptor subfamily 4 group A member 3	0.75	**0.002**	0.395	56%	anti-inflammatory
**Apod**	Apolipoprotein D	0.66	**0.002**	0.358	43%	anti-inflammatory
**Mt3**	Metallothionein 3	0.64	**0.000**	0.079	41%	anti-inflammatory
**S100a1**	S100 calcium binding protein A1	0.56	**0.006**	0.645	31%	inflammatory
**Csf1**	Colony stimulating factor 1	0.50	**0.002**	0.395	25%	anti-inflammatory
**Downregulated genes**						
**Tnc**	Tenascin C	-0.87	**0.002**	0.329	76%	prevents astrogliosis
**Hgf**	Hepatocyte growth factor	-0.83	**0.001**	0.306	69%	prevents astrogliosis
**Ptpn13**	Protein tyrosine phosphatase non-receptor type 13	-0.48	**0.011**	0.777	23%	inflammatory, promotes NF-κB activity

Supplementary Table 8. Upregulated and downregulated inflammation-related genes in microglia.

Gene	Encoded protein	log2 Fold Change	p value	Adjusted p value	Fold change (%)	Potential impact of transcription change
**Upregulated genes**						
**Tnfaip2**	TNF alpha induced protein 2	1.200	**0.031**	0.427	143%	inflammatory
**Ccl3**	C-C motif chemokine ligand 3	1.010	**0.024**	0.382	103%	inflammatory
**Bcl2a1d**	B cell leukemia/lymphoma 2 related protein A1d	1.010	**0.000**	**0.002**	101%	anti-inflammatory
**Bcl2a1b**	B cell leukemia/lymphoma 2 related protein A1b	0.890	**0.000**	**0.002**	79%	anti-inflammatory
**Nr4a3**	Nuclear receptor subfamily 4 group A member 3	0.731	**0.001**	0.064	54%	anti-inflammatory
**Cd83**	CD83	0.710	**0.000**	**0.006**	50%	anti-inflammatory
**Tnf**	Tumor necrosis factor	0.700	**0.000**	**0.036**	49%	inflammatory
**Ccl9**	C-C motif chemokine ligand 9	0.650	**0.002**	0.111	43%	inflammatory
**Gadd45b**	Growth arrest and DNA damage inducible beta	0.680	**0.000**	**0.018**	46%	anti-inflammatory
**Apod**	Apolipoprotein D	0.670	**0.002**	0.095	45%	anti-inflammatory
**Nr4a1**	Nuclear receptor subfamily 4 group A member 1	0.506	**0.001**	0.054	26%	inflammatory
**Cd68**	CD68	0.480	**0.000**	**0.018**	23%	neuroprotective
**Csf1**	Colony stimulating factor 1	0.520	**0.000**	**0.030**	27%	anti-inflammatory
**Ccrl2**	C-C motif chemokine receptor like 2	0.450	**0.001**	0.095	20%	anti-inflammatory
**Downregulated genes**						
**Ccr1**	C-C motif chemokine receptor 1	-1.590	**2E-10**	**4E-07**	253%	anti-inflammatory
**Il7r**	Interleukin 7 receptor	-1.290	**0.013**	0.306	167%	anti-inflammatory
**Thbs1**	Thrombospondin 1	-1.010	**0.000**	**0.008**	102%	inflammatory
**Cd163**	CD163	-0.820	**0.000**	**0.002**	67%	inflammatory
**Tlr4**	Toll like receptor 4	-0.660	**0.000**	**0.008**	44%	anti-inflammatory
**Ccr5**	C-C motif chemokine receptor 5	-0.540	**2E-05**	**4E-03**	29%	neuroprotective
**Tlr7**	Toll like receptor 7	-0.430	**0.000**	**0.032**	19%	anti-inflammatory

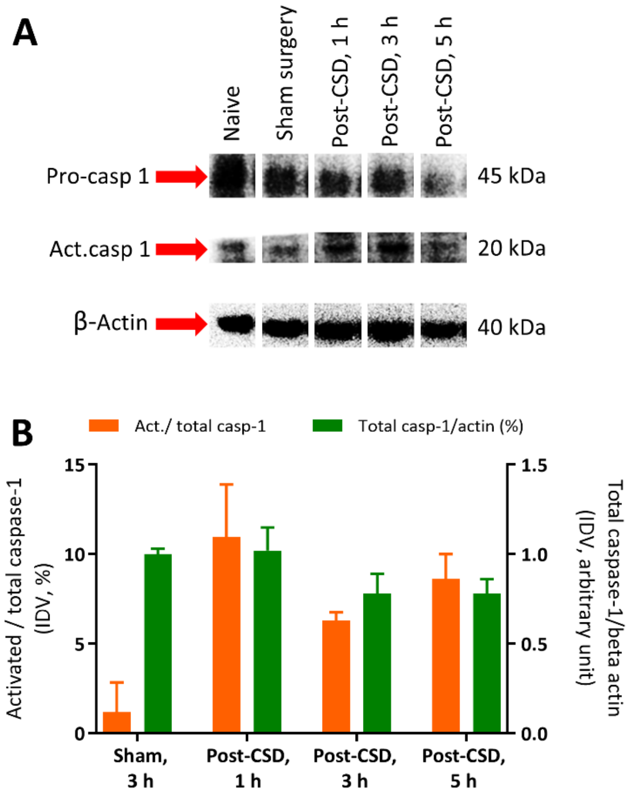

Supplementary Figure 1. Caspase-1 activation exhibits a decreasing trend over the course of 5 hours, as observed by Western blotting. A. Western blotting shows an increase in activated caspase-1 (act. casp-1) following CSD. **B.** The orange columns (left Y-axis) illustrate the active fraction in the total enzyme (active + pro form; right Y-axis), calculated using integrated density values (IDV). To isolate the impact of CSD, the active enzyme fraction in the naïve brain was subtracted from all samples. Active caspase-1 increases after CSD while the total caspase-1 amount remains relatively constant across the time points (green columns, right Y-axis) ($p=0.09$ for activated enzyme ratios; $p=0.29$ for total enzyme amount, Kruskal-Wallis test, $n=3$ mice for each time point). Columns and error bars represent mean and SEM.

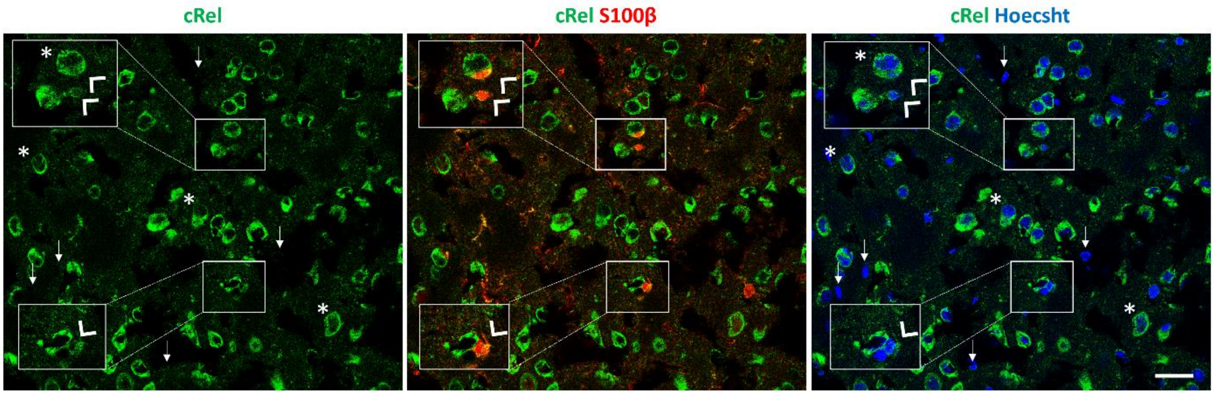

Supplementary Figure 2. Nuclear translocation of NF-κB cRel in astrocytes 24 hours post-CSD. The insets highlight the astrocytes [S100β (+), red, white arrowheads] showing nuclear cRel immunolabeling. Additionally, some neuronal nuclei (*, large nuclei with intense cytoplasmic labeling) also exhibit cRel immunopositivity (see also Figs. 5B and D). In contrast, no nuclear immunoreactivity is observed in the small nuclei of S100β-negative non-neuronal cells (i.e., microglia and oligodendrocytes; arrows). Scale bar: 25 µm.

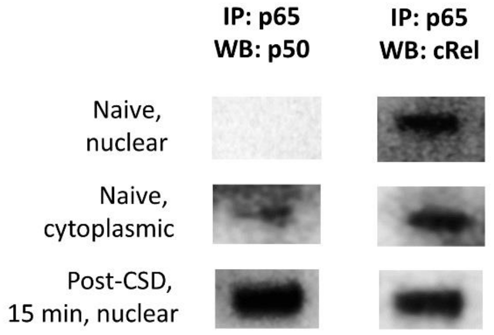

Supplementary Figure 3. Co-immunoprecipitation reveals the presence of p65:p50 and p65:cRel dimers. The dimers were detected in both cytoplasmic and nuclear fractions obtained 15 minutes post-CSD (n=1 mouse per group).

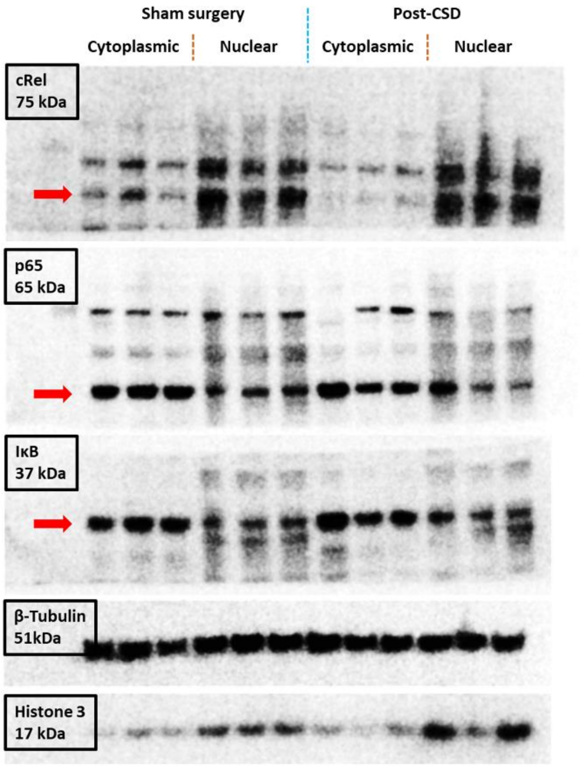

Supplementary Figure 4. Full-length blots of pro- and anti-inflammatory NF-κB subunits p65, cRel, and IκB in both the nucleus and cytoplasm of ipsilateral cortical cells 1-hour after sham surgery or CSD.

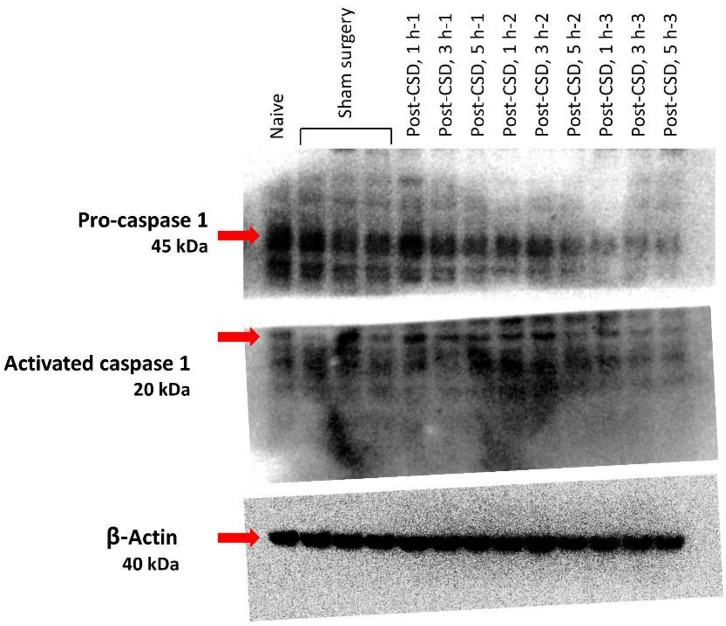

Supplementary Figure 5. Full-length blots of pro- and activated caspase 1 in naïve, 3 hours after sham surgery, and 1, 3, or 5 hours post-CSD.

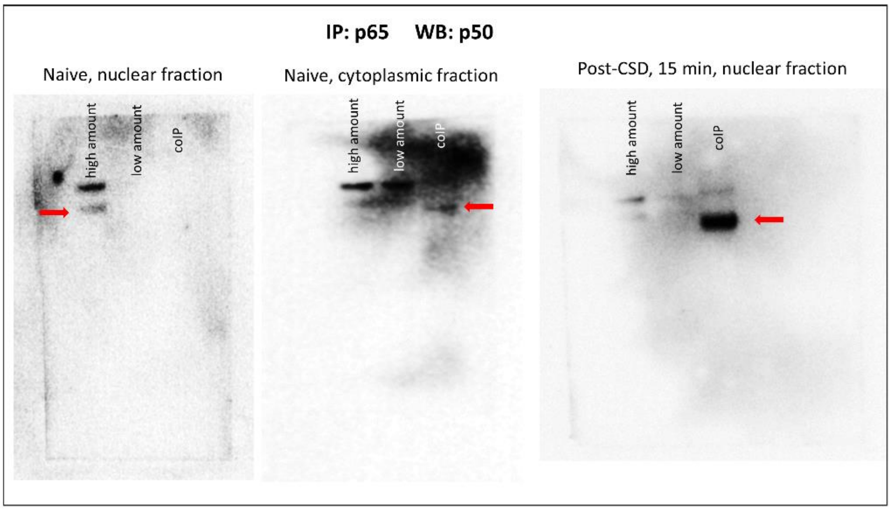

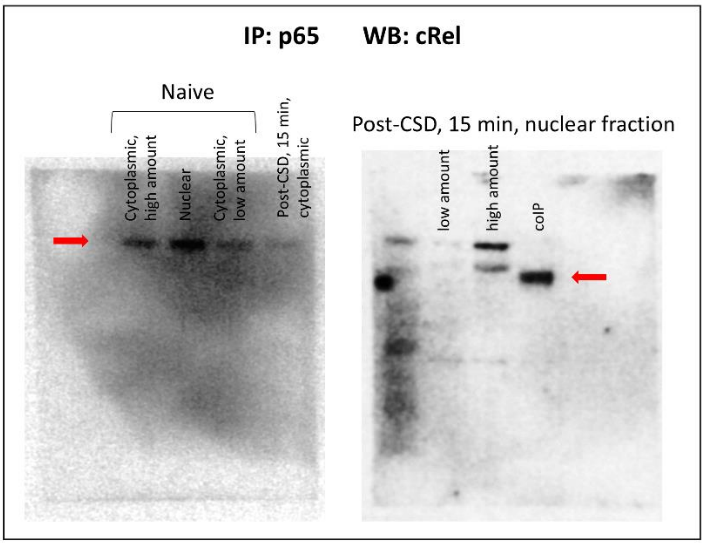

Supplementary Figure 6. Full-length blots of co-immunoprecipitation of p65:p50 and p65:cRel dimers in the nucleus or cytoplasm of ipsilateral cortical cells of naïve brains or 15 minutes post-CSD.
